# Supplementary material for: Longitudinal proteomic profiling of the inflammatory response in dengue patients
Source: PLoS Negl Trop Dis. 2023 Jan 3;17(1):e0011041. doi: 10.1371/journal.pntd.0011041 (PMC9838874; doi:10.1371/journal.pntd.0011041)
Supplement: S1 Table — (DOCX) [file pntd.0011041.s001.docx]

**S1 Table. List of the differentially expressed proteins (DEPs)**

| **Protein** | **UniProt** | **Assay** | **DEPs Acute vs Convalescent** | | | **DEPs Acute vs Controls** | | |
| --- | --- | --- | --- | --- | --- | --- | --- | --- |
|  |  |  | **Log2FC** | ***p*-value** | **FDR** | **Log2FC** | ***p*-value** | **FDR** |
| Adenosine Deaminase | P00813 | ADA | 1.23 | 1.77E-13 | 1.92E-12 | 1.77 | 3.16E-13 | 6.27E-12 |
| Disintegrin and metalloproteinase domain-containing protein 23 | O75077 | ADAM23 | -0.42 | 3.91E-03 | 6.40E-03 | -0.34 | 2.13E-01 | 2.80E-01 |
| Adhesion G protein-coupled receptor E2 | Q9UHX3 | ADGRE2 | 1.53 | 1.29E-15 | 2.06E-14 | 1.95 | 1.64E-14 | 4.61E-13 |
| Advanced Glycosylation End-Product Specific Receptor | Q15109 | AGER | -0.03 | 7.36E-01 | 7.97E-01 | 0.09 | 7.19E-01 | 7.62E-01 |
| Agrin proteoglycan | O00468 | AGRN | 1.17 | 1.61E-10 | 9.38E-10 | 1.83 | 1.30E-09 | 9.53E-09 |
| Agouti-related protein | O00253 | AGRP | 0.58 | 1.17E-02 | 1.81E-02 | 0.93 | 2.15E-02 | 3.43E-02 |
| Aldehyde dehydrogenase | P30838 | ALDH3A1 | 0.07 | 6.05E-01 | 6.64E-01 | 0.70 | 1.27E-02 | 2.17E-02 |
| Ameloblastin | Q9NP70 | AMBN | -0.30 | 4.13E-04 | 7.61E-04 | -0.22 | 2.78E-01 | 3.49E-01 |
| Protein amnionless | Q9BXJ7 | AMN | -0.12 | 3.56E-01 | 4.13E-01 | -0.02 | 9.56E-01 | 9.62E-01 |
| Angiopoietin-1 | Q15389 | ANGPT1 | -0.61 | 2.48E-02 | 3.55E-02 | 0.14 | 7.18E-01 | 7.62E-01 |
| Angiopoietin-related protein 2 | Q9UKU9 | ANGPTL2 | 0.38 | 3.27E-03 | 5.46E-03 | 1.14 | 9.87E-07 | 3.82E-06 |
| Angiopoietin-related protein 4 | Q9BY76 | ANGPTL4 | 0.67 | 2.44E-05 | 5.68E-05 | 0.90 | 8.61E-04 | 1.94E-03 |
| Annexin A11 | P50995 | ANXA11 | 0.03 | 9.21E-01 | 9.44E-01 | 0.14 | 7.49E-01 | 7.89E-01 |
| Rho guanine nucleotide exchange factor 12 | Q9NZN5 | ARHGEF12 | -0.06 | 8.13E-01 | 8.62E-01 | -0.32 | 3.54E-01 | 4.28E-01 |
| ATPase inhibitor, mitochondrial | Q9UII2 | ATP5IF1 | 0.49 | 2.03E-01 | 2.48E-01 | 0.83 | 1.39E-01 | 1.89E-01 |
| Axin-1 | O15169 | AXIN1 | -0.16 | 5.17E-01 | 5.75E-01 | -0.29 | 4.10E-01 | 4.84E-01 |
| Beta-1,4-galactosyltransferase 1 | P15291 | B4GALT1 | 0.67 | 2.80E-04 | 5.45E-04 | 1.11 | 7.04E-04 | 1.59E-03 |
| Transcription regulator protein BACH1 | O14867 | BACH1 | 2.04 | 4.63E-10 | 2.52E-09 | 2.45 | 6.59E-08 | 3.22E-07 |
| B-cell scaffold protein with ankyrin repeats | Q8NDB2 | BANK1 | -0.32 | 3.53E-01 | 4.13E-01 | -0.22 | 6.79E-01 | 7.29E-01 |
| Bcl-2-like protein 11 | O43521 | BCL2L11 | 0.02 | 8.52E-01 | 8.86E-01 | 0.08 | 6.42E-01 | 7.05E-01 |
| Breakpoint cluster region protein | P11274 | BCR | 0.82 | 2.04E-05 | 4.92E-05 | 1.09 | 5.43E-04 | 1.24E-03 |
| BH3-interacting domain death agonist | P55957 | BID | 0.52 | 1.21E-02 | 1.86E-02 | 1.69 | 6.77E-06 | 2.19E-05 |
| Basigin | P35613 | BSG | 0.20 | 7.70E-04 | 1.38E-03 | 0.52 | 7.27E-05 | 1.93E-04 |
| Butyrophilin subfamily 2 member A1 | Q7KYR7 | BTN2A1 | 0.21 | 6.52E-04 | 1.18E-03 | 0.71 | 2.13E-06 | 7.45E-06 |
| Butyrophilin subfamily 3 member A2 | P78410 | BTN3A2 | 0.66 | 3.12E-06 | 9.00E-06 | 1.27 | 2.17E-06 | 7.45E-06 |
| Complement C1q subcomponent subunit A | P02745 | C1QA | 0.71 | 1.15E-12 | 1.14E-11 | 0.73 | 1.33E-06 | 4.99E-06 |
| Caspase-2 | P42575 | CASP2 | -0.01 | 9.51E-01 | 9.63E-01 | 0.24 | 2.68E-01 | 3.40E-01 |
| C-C motif chemokine ligand 11; Eotaxin | P51671 | CCL11 | 0.40 | 6.56E-04 | 1.18E-03 | 0.86 | 1.89E-04 | 4.58E-04 |
| C-C motif chemokine ligand 13; Monocyte chemotactic protein 4 | Q99616 | CCL13 | 0.17 | 4.67E-01 | 5.27E-01 | 0.73 | 2.22E-02 | 3.51E-02 |
| C-C motif chemokine ligand 17 | Q92583 | CCL17 | -1.08 | 1.32E-03 | 2.29E-03 | -0.45 | 3.16E-01 | 3.89E-01 |
| C-C motif chemokine ligand 20; Macrophage inflammatory protein 3 alpha | P78556 | CCL20 | 0.61 | 1.19E-02 | 1.83E-02 | 1.65 | 1.04E-04 | 2.70E-04 |
| C-C motif chemokine ligand 21 | O00585 | CCL21 | 0.51 | 1.29E-04 | 2.62E-04 | 0.67 | 1.16E-02 | 2.02E-02 |
| C-C motif chemokine ligand 22 | O00626 | CCL22 | -0.40 | 6.13E-02 | 8.14E-02 | 0.55 | 1.30E-01 | 1.79E-01 |
| C-C motif chemokine ligand 23 | P55773 | CCL23 | 0.19 | 1.82E-01 | 2.23E-01 | 0.81 | 8.77E-03 | 1.60E-02 |
| C-C motif chemokine ligand 24; Eotaxin-2 | O00175 | CCL24 | -1.08 | 1.28E-06 | 3.93E-06 | -0.94 | 6.11E-03 | 1.14E-02 |
| C-C motif chemokine ligand 25 | O15444 | CCL25 | 1.45 | 1.02E-07 | 3.44E-07 | 1.86 | 2.22E-05 | 6.61E-05 |
| C-C motif chemokine ligand 26; Eotaxin-3 or Macrophage inflammatory protein 4-alpha | Q9Y258 | CCL26 | 0.93 | 1.63E-05 | 3.96E-05 | 1.40 | 1.32E-04 | 3.32E-04 |
| C-C motif chemokine ligand 28 | Q9NRJ3 | CCL28 | 0.44 | 3.13E-02 | 4.40E-02 | 0.30 | 3.46E-01 | 4.19E-01 |
| C-C motif chemokine ligand 3; Macrophage inflammatory protein 1-alpha | P10147 | CCL3 | 2.17 | 3.94E-12 | 3.24E-11 | 2.89 | 2.76E-09 | 1.90E-08 |
| C-C motif chemokine ligand 4; Macrophage inflammatory protein 1-beta | P13236 | CCL4 | 0.43 | 3.27E-02 | 4.58E-02 | 1.15 | 1.69E-03 | 3.66E-03 |
| C-C motif chemokine ligand 7; Monocyte chemotactic protein-3 | P80098 | CCL7 | 2.53 | 1.57E-08 | 6.08E-08 | 2.83 | 2.12E-05 | 6.39E-05 |
| Cellular Communication Network Factor 2 | P29279 | CCN2 | 0.02 | 8.21E-01 | 8.67E-01 | 0.50 | 1.27E-02 | 2.17E-02 |
| CD160 antigen | O95971 | CD160 | -0.17 | 7.62E-02 | 9.88E-02 | 0.49 | 1.15E-02 | 2.02E-02 |
| OX-2 membrane glycoprotein; CD200 | P41217 | CD200 | 0.38 | 6.08E-06 | 1.56E-05 | 0.45 | 2.78E-03 | 5.69E-03 |
| Cell surface glycoprotein CD200 receptor 1 | Q8TD46 | CD200R1 | 0.61 | 4.47E-09 | 2.04E-08 | 1.18 | 3.99E-08 | 2.17E-07 |
| B-cell receptor CD22 | P20273 | CD22 | -0.18 | 3.50E-03 | 5.81E-03 | 0.34 | 2.50E-02 | 3.91E-02 |
| Natural killer cell receptor 2B4 | Q9BZW8 | CD244 | -0.17 | 9.54E-03 | 1.50E-02 | 0.19 | 1.18E-01 | 1.63E-01 |
| CD276 antigen | Q5ZPR3 | CD276 | 0.18 | 1.33E-02 | 2.02E-02 | 0.44 | 1.38E-02 | 2.34E-02 |
| T-cell surface glycoprotein CD4 | P01730 | CD4 | 0.35 | 4.29E-03 | 6.99E-03 | 0.73 | 2.25E-02 | 3.53E-02 |
| Tumor necrosis factor receptor superfamily member 5; CD40 | P25942 | CD40 | 0.72 | 4.82E-07 | 1.55E-06 | 1.04 | 8.51E-05 | 2.22E-04 |
| CD40 ligand | P29965 | CD40LG | -0.43 | 7.99E-02 | 1.03E-01 | -0.39 | 2.43E-01 | 3.15E-01 |
| CD48 antigen | P09326 | CD48 | 1.24 | 1.25E-18 | 7.00E-17 | 1.87 | 5.90E-17 | 2.91E-15 |
| Lymphocyte function-associated antigen 3 | P19256 | CD58 | -0.09 | 9.91E-02 | 1.26E-01 | -0.02 | 8.62E-01 | 8.79E-01 |
| T-cell differentiation antigen CD6 | P30203 | CD6 | -0.18 | 1.62E-02 | 2.39E-02 | 0.33 | 5.26E-02 | 7.64E-02 |
| CD70 antigen | P32970 | CD70 | 2.34 | 2.86E-18 | 1.09E-16 | 2.81 | 2.33E-12 | 3.74E-11 |
| B-cell antigen receptor complex-associated protein beta chain | P40259 | CD79B | 0.51 | 2.93E-04 | 5.64E-04 | 1.15 | 2.21E-04 | 5.26E-04 |
| CD83 antigen | Q01151 | CD83 | 0.39 | 5.04E-05 | 1.10E-04 | 1.02 | 2.02E-06 | 7.28E-06 |
| SLAM family member 5 | Q9UIB8 | CD84 | -0.36 | 8.53E-04 | 1.52E-03 | -0.40 | 1.07E-02 | 1.90E-02 |
| Cell adhesion molecule-related/down-regulated by oncogenes | Q4KMG0 | CDON | -1.83 | 2.52E-19 | 1.70E-17 | -1.35 | 1.31E-08 | 7.89E-08 |
| Corneodesmosin | Q15517 | CDSN | -0.13 | 3.61E-01 | 4.17E-01 | -0.11 | 7.07E-01 | 7.54E-01 |
| Carcinoembryonic antigen related cell adhesion molecule family | Q3KPI0 | CEACAM21 | 0.47 | 8.64E-04 | 1.53E-03 | 1.14 | 1.32E-04 | 3.32E-04 |
| Chordin-like protein 1 | Q9BU40 | CHRDL1 | -0.38 | 1.23E-03 | 2.16E-03 | -0.05 | 7.91E-01 | 8.23E-01 |
| Cytoskeleton-associated protein 4 | Q07065 | CKAP4 | 1.29 | 2.76E-09 | 1.36E-08 | 1.76 | 9.60E-07 | 3.76E-06 |
| Creatine kinase mitochondrial 1A 1B | P12532 | CKMT1A_CKMT1B | -0.01 | 9.96E-01 | 9.96E-01 | 0.19 | 5.87E-01 | 6.59E-01 |
| C-type lectin domain family 4 member A | Q9UMR7 | CLEC4A | -0.62 | 3.68E-08 | 1.38E-07 | -0.92 | 2.10E-06 | 7.45E-06 |
| C-type lectin domain family 4 member C | Q8WTT0 | CLEC4C | -0.10 | 4.28E-01 | 4.84E-01 | 0.29 | 2.60E-01 | 3.32E-01 |
| C-type lectin domain family 4 member D | Q8WXI8 | CLEC4D | 0.01 | 9.55E-01 | 9.64E-01 | 0.50 | 6.80E-02 | 9.80E-02 |
| C-type lectin domain family 4 member G | Q6UXB4 | CLEC4G | 0.07 | 7.05E-01 | 7.66E-01 | 0.03 | 9.39E-01 | 9.47E-01 |
| C-type lectin domain family 7 member A | Q9BXN2 | CLEC7A | 0.58 | 4.38E-06 | 1.21E-05 | 0.88 | 1.74E-04 | 4.25E-04 |
| CAP-Gly domain-containing linker protein 2 | Q9UDT6 | CLIP2 | -0.36 | 3.39E-01 | 4.02E-01 | -0.45 | 4.05E-01 | 4.79E-01 |
| Calsyntenin-2 | Q9H4D0 | CLSTN2 | 0.27 | 2.28E-02 | 3.28E-02 | 0.23 | 3.03E-01 | 3.74E-01 |
| Contactin-associated protein-like 2 | Q9UHC6 | CNTNAP2 | 0.65 | 1.26E-08 | 4.99E-08 | 0.96 | 5.28E-05 | 1.42E-04 |
| Collagen alpha-1(IX) chain | P20849 | COL9A1 | -0.01 | 9.70E-01 | 9.74E-01 | 0.99 | 3.67E-02 | 5.57E-02 |
| Collectin-12 | Q5KU26 | COLEC12 | -0.19 | 1.57E-02 | 2.34E-02 | 0.28 | 1.14E-01 | 1.58E-01 |
| Cysteine rich with EGF like domains 2 | Q6UXH1 | CRELD2 | 0.67 | 5.74E-06 | 1.49E-05 | 1.35 | 7.60E-06 | 2.44E-05 |
| Corticotropin-releasing factor-binding protein | P24387 | CRHBP | -1.49 | 7.31E-11 | 4.65E-10 | -0.64 | 3.94E-02 | 5.86E-02 |
| Cysteine-rich motor neuron 1 protein | Q9NZV1 | CRIM1 | 2.01 | 4.04E-22 | 1.36E-19 | 2.18 | 1.86E-18 | 2.08E-16 |
| Crk-like protein | P46109 | CRKL | 0.34 | 2.80E-01 | 3.39E-01 | 0.54 | 2.39E-01 | 3.10E-01 |
| Cytokine receptor-like factor 1 | O75462 | CRLF1 | 0.55 | 1.02E-06 | 3.20E-06 | 0.56 | 2.97E-03 | 5.97E-03 |
| Macrophage colony-stimulating factor 1 | P09603 | CSF1 | 1.54 | 2.86E-12 | 2.47E-11 | 2.19 | 3.69E-10 | 3.36E-09 |
| Granulocyte colony-stimulating factor | P09919 | CSF3 | 0.29 | 1.16E-01 | 1.46E-01 | 0.21 | 4.99E-01 | 5.75E-01 |
| Cystatin-F | O76096 | CST7 | 1.04 | 3.41E-09 | 1.62E-08 | 1.53 | 1.12E-08 | 6.86E-08 |
| Chymotrypsin-C | Q99895 | CTRC | 0.98 | 2.56E-08 | 9.70E-08 | 1.37 | 6.25E-05 | 1.67E-04 |
| Cathepsin-C; Dipeptidyl peptidase 1 | P53634 | CTSC | 1.94 | 4.99E-15 | 7.31E-14 | 2.62 | 2.19E-10 | 2.11E-09 |
| Cathepsin-O | P43234 | CTSO | 0.90 | 5.62E-08 | 1.99E-07 | 1.18 | 4.52E-06 | 1.51E-05 |
| Coxsackievirus and adenovirus receptor | P78310 | CXADR | 0.43 | 4.00E-04 | 7.40E-04 | 1.25 | 1.44E-06 | 5.32E-06 |
| C-X-C motif chemokine ligand 1 | P09341 | CXCL1 | 0.57 | 7.12E-03 | 1.13E-02 | 1.51 | 4.98E-03 | 9.59E-03 |
| C-X-C motif chemokine ligand 10; Interferon gamma-induced protein | P02778 | CXCL10 | 3.63 | 4.00E-18 | 1.35E-16 | 5.10 | 5.17E-17 | 2.91E-15 |
| C-X-C motif chemokine ligand 12 | P48061 | CXCL12 | 0.51 | 1.44E-02 | 2.16E-02 | 0.35 | 2.85E-01 | 3.55E-01 |
| C-X-C motif chemokine ligand 17 | Q6UXB2 | CXCL17 | 0.22 | 1.35E-01 | 1.68E-01 | 0.03 | 9.38E-01 | 9.47E-01 |
| C-X-C motif chemokine ligand 3 | P19876 | CXCL3 | -0.68 | 3.99E-02 | 5.53E-02 | -0.31 | 5.49E-01 | 6.22E-01 |
| C-X-C motif chemokine ligand 6 | P80162 | CXCL6 | 0.02 | 9.40E-01 | 9.54E-01 | 0.69 | 2.82E-02 | 4.36E-02 |
| C-X-C motif chemokine ligand 8; Interleukin-8 | P10145 | CXCL8 | 1.99 | 1.72E-11 | 1.16E-10 | 3.16 | 6.01E-13 | 1.07E-11 |
| C-X-C motif chemokine ligand 9; Gamma-interferon-induced monokine | Q07325 | CXCL9 | 1.45 | 1.70E-07 | 5.58E-07 | 3.06 | 4.32E-12 | 6.52E-11 |
| Dystroglycan | Q14118 | DAG1 | 0.92 | 5.30E-09 | 2.35E-08 | 1.33 | 1.16E-10 | 1.26E-09 |
| Dual adapter for phosphotyrosine and 3-phosphotyrosine and 3-phosphoinositide | Q9UN19 | DAPP1 | 0.05 | 8.02E-01 | 8.58E-01 | -0.61 | 1.06E-01 | 1.48E-01 |
| Drebrin-like protein | Q9UJU6 | DBNL | -0.07 | 8.63E-01 | 8.95E-01 | 0.35 | 5.00E-01 | 5.75E-01 |
| 2,4-dienoyl-CoA reductase | Q16698 | DECR1 | 1.43 | 2.12E-05 | 5.03E-05 | 1.63 | 2.85E-03 | 5.75E-03 |
| DNA fragmentation factor subunit alpha | O00273 | DFFA | 1.75 | 1.02E-09 | 5.27E-09 | 2.38 | 1.74E-08 | 1.03E-07 |
| DnaJ homolog subfamily A member 2 | O60884 | DNAJA2 | 0.56 | 6.12E-02 | 8.14E-02 | 0.99 | 1.76E-02 | 2.87E-02 |
| Delta and Notch-like epidermal growth factor-related receptor | Q8NFT8 | DNER | -0.01 | 8.09E-01 | 8.60E-01 | 0.12 | 3.62E-01 | 4.34E-01 |
| 2'-deoxynucleoside 5'-phosphate N-hydrolase 1 | O43598 | DNPH1 | 0.96 | 1.70E-04 | 3.41E-04 | 1.36 | 2.79E-04 | 6.54E-04 |
| Inactive dipeptidyl peptidase 10 | Q8N608 | DPP10 | 0.99 | 1.58E-11 | 1.11E-10 | 1.05 | 7.05E-08 | 3.38E-07 |
| Tumor necrosis factor receptor superfamily member EDAR | Q9UNE0 | EDAR | -0.86 | 2.08E-04 | 4.13E-04 | -0.75 | 5.51E-03 | 1.05E-02 |
| Pro-epidermal growth factor | P01133 | EGF | -0.65 | 1.05E-01 | 1.32E-01 | 0.35 | 5.50E-01 | 6.22E-01 |
| Egl nine homolog 1 | Q9GZT9 | EGLN1 | 1.64 | 3.85E-08 | 1.43E-07 | 2.39 | 1.57E-07 | 6.94E-07 |
| Eukaryotic translation initiation factor 4 gamma 1 | Q04637 | EIF4G1 | 2.36 | 3.82E-09 | 1.79E-08 | 3.04 | 1.04E-08 | 6.48E-08 |
| Protein enabled homolog | Q8N8S7 | ENAH | -0.04 | 8.34E-01 | 8.75E-01 | 0.06 | 8.64E-01 | 8.79E-01 |
| Ectonucleotide pyrophosphatase/phosphodiesterase family member 5 | Q9UJA9 | ENPP5 | 0.02 | 7.41E-01 | 8.01E-01 | -0.31 | 3.87E-02 | 5.83E-02 |
| Ectonucleotide pyrophosphatase/phosphodiesterase family member 7 | Q6UWV6 | ENPP7 | 1.49 | 2.33E-08 | 8.92E-08 | 2.56 | 3.00E-08 | 1.68E-07 |
| Epithelial cell adhesion molecule | P16422 | EPCAM | 0.10 | 2.93E-01 | 3.53E-01 | -0.83 | 1.12E-02 | 1.97E-02 |
| Ephrin type-A receptor 1 | P21709 | EPHA1 | 0.23 | 1.46E-02 | 2.19E-02 | 0.58 | 3.80E-05 | 1.06E-04 |
| Erythropoietin | P01588 | EPO | 0.52 | 1.36E-02 | 2.06E-02 | 1.02 | 1.19E-02 | 2.05E-02 |
| Receptor tyrosine-protein kinase erbB-3 | P21860 | ERBB3 | 0.67 | 6.59E-12 | 4.93E-11 | 1.06 | 3.10E-11 | 4.02E-10 |
| Endothelial cell-specific molecule 1 | Q9NQ30 | ESM1 | 3.62 | 1.52E-21 | 1.44E-19 | 3.20 | 3.01E-13 | 6.27E-12 |
| Proteinase-activated receptor 1 | P25116 | F2R | 0.06 | 8.08E-01 | 8.60E-01 | 0.43 | 2.77E-01 | 3.48E-01 |
| Fatty acid-binding protein | P07148 | FABP1 | 2.05 | 9.71E-08 | 3.30E-07 | 3.54 | 5.92E-08 | 3.02E-07 |
| Fatty acid-binding protein 9 | Q0Z7S8 | FABP9 | 0.56 | 3.30E-04 | 6.29E-04 | 0.77 | 1.59E-02 | 2.61E-02 |
| Tumor necrosis factor ligand superfamily member 6 | P48023 | FASLG | 0.55 | 1.26E-05 | 3.08E-05 | 0.72 | 2.31E-03 | 4.87E-03 |
| Immunoglobulin alpha Fc receptor | P24071 | FCAR | 0.84 | 2.40E-10 | 1.35E-09 | 1.50 | 1.12E-07 | 5.18E-07 |
| Fc receptor-like protein 2 | Q96LA5 | FCRL2 | 0.36 | 1.11E-03 | 1.96E-03 | 0.82 | 1.60E-03 | 3.51E-03 |
| Fc receptor-like protein 3 | Q96P31 | FCRL3 | -0.19 | 2.19E-02 | 3.16E-02 | 0.40 | 4.82E-02 | 7.09E-02 |
| Fc receptor-like protein 6 | Q6DN72 | FCRL6 | 0.44 | 4.74E-06 | 1.27E-05 | 1.09 | 1.40E-05 | 4.32E-05 |
| Fibroblast growth factor 19 | O95750 | FGF19 | -0.32 | 6.77E-02 | 8.91E-02 | 0.04 | 9.05E-01 | 9.18E-01 |
| Fibroblast growth factor 5 | P12034 | FGF5 | 0.13 | 3.19E-01 | 3.83E-01 | 0.18 | 4.77E-01 | 5.52E-01 |
| Mitochondrial fission 1 protein | Q9Y3D6 | FIS1 | -0.24 | 4.20E-01 | 4.78E-01 | 0.27 | 5.17E-01 | 5.91E-01 |
| Fms-related tyrosine kinase 3 ligand | P49771 | FLT3LG | 0.05 | 5.79E-01 | 6.37E-01 | 0.11 | 5.82E-01 | 6.56E-01 |
| Forkhead box protein O1 | Q12778 | FOXO1 | 0.82 | 5.17E-04 | 9.41E-04 | 0.96 | 1.59E-02 | 2.61E-02 |
| Follistatin | P19883 | FST | 0.78 | 5.75E-05 | 1.23E-04 | 0.73 | 5.23E-02 | 7.63E-02 |
| Follistatin-related protein 3 | O95633 | FSTL3 | 0.63 | 6.23E-06 | 1.59E-05 | 0.98 | 2.20E-04 | 5.26E-04 |
| FXYD domain-containing ion transport regulator 5 | Q96DB9 | FXYD5 | 0.43 | 4.71E-05 | 1.03E-04 | 0.17 | 3.22E-01 | 3.94E-01 |
| Galanin peptides | P22466 | GAL | -1.43 | 1.57E-08 | 6.08E-08 | -1.60 | 4.51E-04 | 1.04E-03 |
| Polypeptide N-acetylgalactosaminyltransferase 3 | Q14435 | GALNT3 | 1.36 | 2.36E-11 | 1.56E-10 | 1.72 | 6.45E-08 | 3.19E-07 |
| Guanylate-binding protein 2 | P32456 | GBP2 | 3.19 | 5.46E-12 | 4.18E-11 | 4.32 | 1.55E-10 | 1.53E-09 |
| Glyoxalase domain containing 4 | Q9HC38 | GLOD4 | 0.67 | 1.07E-04 | 2.23E-04 | 1.10 | 1.15E-04 | 2.95E-04 |
| GMP reductase 1 | P36959 | GMPR | 0.49 | 4.02E-02 | 5.55E-02 | 0.08 | 8.40E-01 | 8.63E-01 |
| Golgi-associated PDZ and coiled-coil motif-containing protein | Q9HD26 | GOPC | 1.46 | 2.43E-06 | 7.17E-06 | 1.52 | 1.65E-04 | 4.09E-04 |
| Granzyme A | P12544 | GZMA | 1.95 | 9.07E-14 | 1.09E-12 | 2.77 | 1.29E-10 | 1.36E-09 |
| Granzyme B | P10144 | GZMB | 4.58 | 4.18E-16 | 7.42E-15 | 5.77 | 1.83E-14 | 4.73E-13 |
| Hematopoietic lineage cell-specific protein | P14317 | HCLS1 | 1.46 | 4.55E-06 | 1.23E-05 | 1.79 | 4.90E-04 | 1.12E-03 |
| Protein HEXIM1 | O94992 | HEXIM1 | 1.66 | 1.32E-07 | 4.39E-07 | 1.93 | 7.87E-06 | 2.50E-05 |
| Hepatocyte growth factor | P14210 | HGF | 1.80 | 1.90E-16 | 4.01E-15 | 2.44 | 4.71E-13 | 8.82E-12 |
| HLA class II histocompatibility antigen, DR alpha chain | P01903 | HLA-DRA | 0.65 | 2.08E-06 | 6.20E-06 | 1.59 | 5.90E-07 | 2.42E-06 |
| HLA class I histocompatibility antigen, alpha chain E | P13747 | HLA-E | 0.35 | 3.45E-04 | 6.50E-04 | 0.50 | 1.17E-02 | 2.02E-02 |
| Hippocalcin-like protein 1 | P37235 | HPCAL1 | 1.43 | 1.23E-06 | 3.81E-06 | 1.73 | 1.09E-05 | 3.40E-05 |
| Corticosteroid 11-beta-dehydrogenase isozyme 1 | P28845 | HSD11B1 | 1.30 | 4.08E-12 | 3.27E-11 | 1.52 | 2.61E-09 | 1.83E-08 |
| Heat shock 70 kDa protein 1A | P0DMV8 | HSPA1A | 2.42 | 1.55E-10 | 9.17E-10 | 3.01 | 1.07E-09 | 8.42E-09 |
| Islet cell autoantigen 1 | Q05084 | ICA1 | 0.20 | 3.60E-01 | 4.17E-01 | 0.36 | 2.02E-01 | 2.67E-01 |
| Intercellular adhesion molecule 4 | Q14773 | ICAM4 | -0.18 | 5.93E-02 | 7.92E-02 | -0.10 | 6.01E-01 | 6.66E-01 |
| Iduronate 2-sulfatase | P22304 | IDS | 0.09 | 5.16E-02 | 7.01E-02 | 0.09 | 2.83E-01 | 3.53E-01 |
| Interferon gamma | P01579 | IFNG | 2.28 | 4.46E-06 | 1.21E-05 | 3.46 | 2.65E-05 | 7.62E-05 |
| Interferon gamma receptor 1 | P15260 | IFNGR1 | 0.15 | 5.84E-03 | 9.33E-03 | 0.33 | 1.94E-02 | 3.13E-02 |
| Interferon lambda receptor 1 | Q8IU57 | IFNLR1 | 1.17 | 4.81E-14 | 6.01E-13 | 1.69 | 7.03E-13 | 1.18E-11 |
| NF-kappa-B essential modulator | Q9Y6K9 | IKBKG | 0.67 | 1.29E-02 | 1.97E-02 | 1.21 | 2.51E-03 | 5.18E-03 |
| Interleukin-10 | P22301 | IL10 | 3.85 | 2.95E-13 | 3.11E-12 | 4.11 | 3.73E-09 | 2.46E-08 |
| Interleukin-10 receptor subunit alpha | Q13651 | IL10RA | 0.34 | 4.64E-05 | 1.02E-04 | 0.04 | 8.11E-01 | 8.39E-01 |
| Interleukin-10 receptor subunit beta | Q08334 | IL10RB | 0.06 | 3.66E-01 | 4.21E-01 | 0.52 | 2.55E-03 | 5.24E-03 |
| Interleukin-12 subunit beta | P29460 | IL12B | -0.01 | 9.30E-01 | 9.47E-01 | 0.46 | 7.15E-02 | 1.02E-01 |
| Interleukin-12 receptor subunit beta-1 | P42701 | IL12RB1 | 1.04 | 2.86E-12 | 2.47E-11 | 1.76 | 1.29E-13 | 3.10E-12 |
| Interleukin-15 | P40933 | IL15 | 0.68 | 3.51E-04 | 6.58E-04 | 0.78 | 1.45E-02 | 2.44E-02 |
| Interleukin 15 receptor subunit alpha | Q13261 | IL15RA | 0.63 | 6.14E-08 | 2.13E-07 | 1.26 | 1.82E-09 | 1.30E-08 |
| Pro-interleukin-16 | Q14005 | IL16 | 0.71 | 1.61E-06 | 4.88E-06 | 1.80 | 1.20E-09 | 9.16E-09 |
| Interleukin-17A | Q16552 | IL17A | 0.20 | 4.45E-02 | 6.10E-02 | 0.24 | 1.47E-01 | 1.99E-01 |
| Interleukin-17C | Q9P0M4 | IL17C | 0.70 | 1.41E-04 | 2.85E-04 | 1.39 | 2.21E-04 | 5.26E-04 |
| Interleukin-17D | Q8TAD2 | IL17D | 0.11 | 4.27E-01 | 4.84E-01 | -0.30 | 2.49E-01 | 3.21E-01 |
| Interleukin-17F | Q96PD4 | IL17F | 0.29 | 4.89E-03 | 7.88E-03 | 0.12 | 6.34E-01 | 6.99E-01 |
| Interleukin-17 receptor B | Q9NRM6 | IL17RB | 1.40 | 1.16E-14 | 1.63E-13 | 1.86 | 6.79E-11 | 7.89E-10 |
| Interleukin-18 | Q14116 | IL18 | 0.70 | 1.03E-10 | 6.18E-10 | 1.28 | 1.46E-07 | 6.56E-07 |
| Interleukin-18 receptor 1 | Q13478 | IL18R1 | 1.84 | 1.32E-21 | 1.44E-19 | 2.54 | 2.48E-18 | 2.09E-16 |
| Interleukin-1 beta | P01584 | IL1B | 0.31 | 3.55E-01 | 4.13E-01 | 1.05 | 1.17E-02 | 2.02E-02 |
| Interleukin-1 receptor type 2 | P27930 | IL1R2 | -0.30 | 3.50E-05 | 7.98E-05 | -0.20 | 1.42E-01 | 1.93E-01 |
| Interleukin-1 receptor-like 2 | Q9HB29 | IL1RL2 | 0.42 | 5.60E-05 | 1.20E-04 | 0.32 | 1.19E-01 | 1.64E-01 |
| Interleukin-1 receptor antagonist protein | P18510 | IL1RN | 1.83 | 4.54E-08 | 1.63E-07 | 2.24 | 1.50E-05 | 4.60E-05 |
| Interleukin-20 receptor subunit alpha | Q9UHF4 | IL20RA | 0.11 | 1.43E-01 | 1.78E-01 | 0.29 | 1.64E-01 | 2.20E-01 |
| Interleukin-22 receptor subunit alpha-1 | Q8N6P7 | IL22RA1 | 0.40 | 2.90E-04 | 5.62E-04 | 0.45 | 7.38E-03 | 1.36E-02 |
| Interleukin-32 | P24001 | IL32 | -0.68 | 4.40E-06 | 1.21E-05 | 0.40 | 8.96E-02 | 1.26E-01 |
| Interleukin-33 | O95760 | IL33 | 2.54 | 1.18E-08 | 4.74E-08 | 2.42 | 2.40E-04 | 5.65E-04 |
| Interleukin-3 receptor subunit alpha | P26951 | IL3RA | 0.05 | 4.97E-01 | 5.57E-01 | 0.39 | 2.42E-03 | 5.03E-03 |
| Interleukin-4 receptor subunit alpha | P24394 | IL4R | 1.31 | 6.13E-08 | 2.13E-07 | 1.60 | 3.17E-05 | 8.99E-05 |
| Interleukin-5 receptor subunit alpha | Q01344 | IL5RA | 1.54 | 1.29E-16 | 3.10E-15 | 1.69 | 6.38E-07 | 2.56E-06 |
| Interleukin-6 | P05231 | IL6 | 1.37 | 2.94E-06 | 8.55E-06 | 1.99 | 2.63E-05 | 7.62E-05 |
| Interleukin-7 | P13232 | IL7 | -0.43 | 2.09E-02 | 3.03E-02 | 0.32 | 3.68E-01 | 4.40E-01 |
| Interleukin-1 receptor-associated kinase 1 | P51617 | IRAK1 | 0.63 | 1.31E-03 | 2.28E-03 | 0.68 | 2.85E-02 | 4.39E-02 |
| Interleukin-1 receptor-associated kinase 4 | Q9NWZ3 | IRAK4 | 1.12 | 2.18E-04 | 4.26E-04 | 0.99 | 3.40E-02 | 5.21E-02 |
| Isthmin-1 | B1AKI9 | ISM1 | 0.36 | 1.77E-02 | 2.58E-02 | 0.51 | 7.50E-02 | 1.07E-01 |
| Integrin alpha-11 | Q9UKX5 | ITGA11 | 0.03 | 7.87E-01 | 8.45E-01 | -0.18 | 2.97E-01 | 3.68E-01 |
| Integrin beta-6 | P18564 | ITGB6 | -0.38 | 4.65E-05 | 1.02E-04 | -0.38 | 1.41E-02 | 2.37E-02 |
| Integral membrane protein 2A | O43736 | ITM2A | 0.22 | 2.46E-01 | 2.99E-01 | -0.18 | 5.17E-01 | 5.91E-01 |
| Immunoglobulin J chain | P01591 | JCHAIN | 0.19 | 6.24E-02 | 8.24E-02 | 0.27 | 2.72E-01 | 3.44E-01 |
| Transcription factor AP-1 | P05412 | JUN | 1.03 | 5.49E-07 | 1.74E-06 | 1.29 | 1.56E-05 | 4.74E-05 |
| Killer cell lectin-like receptor subfamily B member 1 | Q12918 | KLRB1 | 0.16 | 5.21E-02 | 7.06E-02 | 1.12 | 4.43E-08 | 2.33E-07 |
| Natural killer cells antigen CD94 | Q13241 | KLRD1 | 0.54 | 3.48E-06 | 9.87E-06 | 1.33 | 5.96E-07 | 2.42E-06 |
| Keratin, type I cytoskeletal 19 | P08727 | KRT19 | 0.13 | 3.36E-01 | 4.01E-01 | 1.20 | 2.51E-05 | 7.37E-05 |
| Kynureninase | Q16719 | KYNU | 2.02 | 1.38E-16 | 3.10E-15 | 2.33 | 1.30E-09 | 9.53E-09 |
| Leukocyte-associated immunoglobulin-like receptor 1 | Q6GTX8 | LAIR1 | 0.82 | 8.87E-09 | 3.65E-08 | 1.58 | 6.95E-09 | 4.45E-08 |
| Laminin subunit alpha-4 | Q16363 | LAMA4 | -0.45 | 1.09E-04 | 2.26E-04 | -0.07 | 6.60E-01 | 7.19E-01 |
| Lysosome-associated membrane glycoprotein 3 | Q9UQV4 | LAMP3 | 2.47 | 1.74E-17 | 4.88E-16 | 3.92 | 1.43E-24 | 4.81E-22 |
| Cytosol aminopeptidase | P28838 | LAP3 | 2.34 | 4.32E-12 | 3.39E-11 | 3.08 | 1.04E-09 | 8.37E-09 |
| Linker for activation of T cells family member 1 | O43561 | LAT | 0.23 | 4.01E-01 | 4.58E-01 | 0.12 | 7.52E-01 | 7.89E-01 |
| Galectin-4 | P56470 | LGALS4 | -0.02 | 9.04E-01 | 9.29E-01 | 0.38 | 2.55E-01 | 3.27E-01 |
| Galectin-9 | O00182 | LGALS9 | 1.27 | 5.07E-16 | 8.54E-15 | 2.18 | 3.95E-16 | 1.33E-14 |
| Legumain | Q99538 | LGMN | 0.68 | 7.82E-05 | 1.64E-04 | 1.21 | 5.20E-05 | 1.41E-04 |
| Phospholysine phosphohistidine inorganic pyrophosphate phosphatase | Q9H008 | LHPP | 1.85 | 9.66E-11 | 5.92E-10 | 2.48 | 4.29E-08 | 2.30E-07 |
| Leukemia inhibitory factor receptor | P42702 | LIFR | 0.86 | 1.24E-11 | 8.87E-11 | 1.01 | 2.01E-07 | 8.66E-07 |
| Leukocyte immunoglobulin-like receptor subfamily B member 4 | Q8NHJ6 | LILRB4 | 1.59 | 1.98E-12 | 1.86E-11 | 1.93 | 2.41E-07 | 1.03E-06 |
| Leucine rich repeat neuronal 1 | Q6UXK5 | LRRN1 | -0.64 | 5.66E-10 | 3.03E-09 | -0.82 | 5.68E-06 | 1.86E-05 |
| Lymphocyte-specific protein 1 | P33241 | LSP1 | 0.87 | 7.78E-05 | 1.64E-04 | 1.75 | 1.03E-06 | 3.93E-06 |
| Lymphotoxin-alpha | P01374 | LTA | 0.55 | 3.94E-06 | 1.11E-05 | 0.76 | 3.47E-04 | 8.07E-04 |
| Tumor necrosis factor receptor superfamily member 3 | P36941 | LTBR | 0.54 | 6.85E-06 | 1.71E-05 | 1.08 | 8.85E-06 | 2.79E-05 |
| Lymphocyte antigen 6D | Q14210 | LY6D | -0.08 | 3.54E-01 | 4.13E-01 | 0.36 | 6.20E-02 | 8.97E-02 |
| Lymphocyte antigen 75 | O60449 | LY75 | 0.13 | 3.05E-02 | 4.31E-02 | 0.42 | 5.02E-03 | 9.61E-03 |
| T-lymphocyte surface antigen Ly-9 | Q9HBG7 | LY9 | 0.40 | 6.52E-09 | 2.74E-08 | 0.97 | 3.97E-08 | 2.17E-07 |
| Mesencephalic astrocyte-derived neurotrophic factor | P55145 | MANF | -0.19 | 6.38E-01 | 6.98E-01 | 0.27 | 6.46E-01 | 7.07E-01 |
| Dual specificity mitogen-activated protein kinase kinase 6 | P52564 | MAP2K6 | 0.08 | 7.79E-01 | 8.38E-01 | 0.12 | 8.00E-01 | 8.30E-01 |
| Mitogen-activated protein kinase 9 | P45984 | MAPK9 | 1.58 | 7.44E-08 | 2.56E-07 | 2.32 | 1.85E-07 | 8.11E-07 |
| Matrilin-2 | O00339 | MATN2 | 0.34 | 1.70E-03 | 2.92E-03 | 0.53 | 5.54E-03 | 1.05E-02 |
| Multiple epidermal growth factor-like domains protein 10 | Q96KG7 | MEGF10 | -0.10 | 1.46E-01 | 1.81E-01 | -0.09 | 6.74E-01 | 7.26E-01 |
| Matrix extracellular phosphoglycoprotein | Q9NQ76 | MEPE | 0.49 | 8.69E-03 | 1.38E-02 | 0.31 | 2.27E-01 | 2.96E-01 |
| Tyrosine-protein kinase Mer | Q12866 | MERTK | 0.50 | 5.07E-06 | 1.34E-05 | 0.88 | 2.28E-05 | 6.74E-05 |
| Methionine aminopeptidase 1D | Q6UB28 | METAP1D | 1.51 | 4.52E-05 | 1.01E-04 | 1.72 | 7.92E-05 | 2.09E-04 |
| Monoglyceride lipase | Q99685 | MGLL | -0.48 | 1.17E-01 | 1.46E-01 | -0.23 | 5.91E-01 | 6.60E-01 |
| Methylated-DNA-protein-cysteine methyltransferase | P16455 | MGMT | 1.29 | 1.26E-04 | 2.58E-04 | 1.44 | 9.12E-04 | 2.03E-03 |
| MHC class I chain-related protein A and B | Q29980_Q29983 | MICB_MICA | 0.65 | 1.55E-07 | 5.12E-07 | 0.55 | 3.41E-01 | 4.15E-01 |
| Allergin-1 | Q7Z6M3 | MILR1 | 0.53 | 4.47E-04 | 8.18E-04 | 0.62 | 3.95E-02 | 5.86E-02 |
| Promotilin | P12872 | MLN | -0.36 | 8.36E-02 | 1.08E-01 | 0.18 | 6.65E-01 | 7.21E-01 |
| Matrix Metallopeptidase 1 | P03956 | MMP1 | 0.88 | 3.18E-03 | 5.33E-03 | 1.54 | 1.92E-03 | 4.07E-03 |
| Matrix Metallopeptidase 10 | P09238 | MMP10 | 0.77 | 6.65E-06 | 1.67E-05 | 0.61 | 8.01E-02 | 1.13E-01 |
| Megakaryocyte and platelet inhibitory receptor G6b | O95866 | MPIG6B | -0.84 | 2.51E-02 | 3.59E-02 | -0.24 | 6.65E-01 | 7.21E-01 |
| Mevalonate kinase | Q03426 | MVK | 1.23 | 2.09E-05 | 4.99E-05 | 1.40 | 3.56E-03 | 7.10E-03 |
| Marginal zone B- and B1-cell-specific protein | Q8WU39 | MZB1 | 1.30 | 1.91E-10 | 1.09E-09 | 2.14 | 1.51E-10 | 1.53E-09 |
| Nibrin | O60934 | NBN | 1.69 | 5.01E-06 | 1.33E-05 | 3.05 | 4.67E-10 | 4.04E-09 |
| Neutrophil cytosol factor 2 | P19878 | NCF2 | 0.55 | 8.99E-02 | 1.15E-01 | 1.53 | 1.79E-03 | 3.87E-03 |
| Cytoplasmic protein NCK2 | O43639 | NCK2 | -0.33 | 8.80E-02 | 1.13E-01 | -0.62 | 3.90E-02 | 5.84E-02 |
| Natural cytotoxicity triggering receptor 1 | O76036 | NCR1 | 0.71 | 2.43E-12 | 2.21E-11 | 1.20 | 2.54E-08 | 1.45E-07 |
| Protein kinase C-binding protein NELL2 | Q99435 | NELL2 | 1.54 | 2.91E-18 | 1.09E-16 | 1.86 | 3.25E-16 | 1.22E-14 |
| Neurofascin | O94856 | NFASC | 1.12 | 3.58E-17 | 9.28E-16 | 1.45 | 3.01E-16 | 1.22E-14 |
| Nuclear factor of activated T-cells, cytoplasmic 1 | O95644 | NFATC1 | 0.02 | 9.29E-01 | 9.47E-01 | 0.26 | 3.56E-01 | 4.29E-01 |
| Nuclear factor of activated T-cells, cytoplasmic 3 | Q12968 | NFATC3 | 0.81 | 4.17E-05 | 9.37E-05 | 0.88 | 9.00E-03 | 1.63E-02 |
| Nucleoside diphosphate kinase 3 | Q13232 | NME3 | 0.05 | 5.17E-01 | 5.75E-01 | 0.12 | 4.16E-01 | 4.87E-01 |
| C-type natriuretic peptide | P23582 | NPPC | -1.42 | 2.81E-06 | 8.24E-06 | -0.95 | 2.23E-02 | 3.51E-02 |
| Neurturin | Q99748 | NRTN | 0.34 | 3.50E-02 | 4.87E-02 | 0.55 | 4.26E-02 | 6.29E-02 |
| Cytosolic 5'-nucleotidase 3A | Q9H0P0 | NT5C3A | 0.54 | 7.53E-02 | 9.80E-02 | 1.00 | 2.54E-02 | 3.94E-02 |
| Neurotrophin-3 | P20783 | NTF3 | -0.26 | 1.58E-02 | 2.34E-02 | 0.08 | 6.73E-01 | 7.26E-01 |
| NEDD8 ultimate buster 1 | Q9Y5A7 | NUB1 | 1.21 | 7.12E-06 | 1.76E-05 | 1.51 | 2.94E-05 | 8.39E-05 |
| Nuclear migration protein nudC | Q9Y266 | NUDC | 1.60 | 2.55E-10 | 1.41E-09 | 2.13 | 2.98E-09 | 2.01E-08 |
| Osteomodulin | Q99983 | OMD | 1.00 | 3.37E-13 | 3.44E-12 | 1.32 | 9.58E-11 | 1.08E-09 |
| Osteoclast-associated immunoglobulin-like receptor | Q8IYS5 | OSCAR | 0.79 | 3.29E-14 | 4.43E-13 | 1.47 | 4.47E-11 | 5.53E-10 |
| Oncostatin-M | P13725 | OSM | 0.71 | 2.69E-02 | 3.83E-02 | 1.81 | 4.26E-06 | 1.44E-05 |
| Pappalysin-1 | Q13219 | PAPPA | 1.55 | 3.45E-11 | 2.23E-10 | 1.59 | 4.94E-08 | 2.56E-07 |
| Poly [ADP-ribose] polymerase 1 | P09874 | PARP1 | 1.73 | 2.96E-05 | 6.78E-05 | 2.48 | 2.03E-06 | 7.28E-06 |
| Protocadherin-1 | Q08174 | PCDH1 | 0.37 | 6.66E-06 | 1.67E-05 | 0.47 | 1.92E-03 | 4.07E-03 |
| Platelet-derived growth factor subunit B | P01127 | PDGFB | -1.01 | 3.74E-03 | 6.18E-03 | -0.15 | 7.62E-01 | 7.96E-01 |
| PDZ and LIM domain protein 7 | Q9NR12 | PDLIM7 | -0.46 | 2.77E-01 | 3.36E-01 | 0.13 | 8.27E-01 | 8.52E-01 |
| Placenta growth factor | P49763 | PGF | 0.40 | 3.37E-04 | 6.39E-04 | 0.68 | 2.39E-03 | 5.01E-03 |
| Phosphoinositide 3-kinase adapter protein 1 | Q6ZUJ8 | PIK3AP1 | 1.77 | 1.01E-08 | 4.11E-08 | 2.46 | 3.56E-07 | 1.50E-06 |
| Pyruvate kinase PKLR | P30613 | PKLR | 1.48 | 4.08E-08 | 1.48E-07 | 2.22 | 5.67E-07 | 2.36E-06 |
| Cytosolic phospholipase A2 | P47712 | PLA2G4A | 0.03 | 8.78E-01 | 9.08E-01 | 0.04 | 8.56E-01 | 8.77E-01 |
| Urokinase plasminogen activator surface receptor | Q03405 | PLAUR | 1.02 | 3.46E-12 | 2.91E-11 | 1.45 | 4.79E-10 | 4.04E-09 |
| Plexin-A4 | Q9HCM2 | PLXNA4 | -0.62 | 2.77E-03 | 4.67E-03 | -0.79 | 5.81E-03 | 1.09E-02 |
| Pancreatic Lipase Related Protein 2 | P54317 | PNLIPRP2 | 0.95 | 9.88E-03 | 1.55E-02 | -0.72 | 5.96E-01 | 6.62E-01 |
| Polyribonucleotide nucleotidyltransferase 1 | Q8TCS8 | PNPT1 | 1.12 | 6.52E-05 | 1.38E-04 | 1.08 | 1.06E-02 | 1.90E-02 |
| Serum paraoxonase/lactonase 3 | Q15166 | PON3 | -0.27 | 1.71E-03 | 2.92E-03 | -0.65 | 4.51E-03 | 8.78E-03 |
| Neurabin-2 | Q96SB3 | PPP1R9B | 0.70 | 1.56E-02 | 2.33E-02 | 0.44 | 2.76E-01 | 3.48E-01 |
| Thioredoxin-dependent peroxide reductase, mitochondrial | P30048 | PRDX3 | 1.62 | 5.62E-06 | 1.47E-05 | 2.05 | 1.18E-06 | 4.45E-06 |
| Peroxiredoxin-5, mitochondrial | P30044 | PRDX5 | 0.55 | 4.78E-02 | 6.53E-02 | 1.44 | 1.12E-03 | 2.46E-03 |
| Prolactin regulatory element-binding protein | Q9HCU5 | PREB | 0.39 | 3.82E-03 | 6.27E-03 | 0.50 | 1.09E-02 | 1.94E-02 |
| Prolargin | P51888 | PRELP | -0.05 | 6.49E-01 | 7.08E-01 | -0.17 | 4.48E-01 | 5.23E-01 |
| 5'-AMP-activated protein kinase subunit beta-1 | Q9Y478 | PRKAB1 | 0.01 | 9.71E-01 | 9.74E-01 | 0.32 | 1.72E-01 | 2.30E-01 |
| Prokineticin-1 | P58294 | PROK1 | 0.53 | 2.96E-04 | 5.67E-04 | 0.78 | 1.00E-02 | 1.81E-02 |
| Prostasin | Q16651 | PRSS8 | -0.32 | 1.64E-03 | 2.83E-03 | 0.11 | 5.48E-01 | 6.22E-01 |
| PC4 and SFRS1-interacting protein | O75475 | PSIP1 | 1.54 | 1.14E-04 | 2.35E-04 | 3.38 | 3.63E-10 | 3.36E-09 |
| Proteasome assembly chaperone 3 | Q9BT73 | PSMG3 | 0.89 | 4.46E-06 | 1.21E-05 | 0.87 | 1.89E-02 | 3.07E-02 |
| Persephin | O60542 | PSPN | -0.93 | 1.68E-12 | 1.61E-11 | -0.77 | 3.64E-02 | 5.55E-02 |
| Parathyroid hormone/parathyroid hormone-related peptide receptor | Q03431 | PTH1R | -0.07 | 5.57E-01 | 6.17E-01 | 0.09 | 6.32E-01 | 6.98E-01 |
| Tyrosine-protein phosphatase non-receptor type 6 | P29350 | PTPN6 | 0.72 | 2.84E-02 | 4.02E-02 | 0.26 | 5.89E-01 | 6.60E-01 |
| Receptor-type tyrosine-protein phosphatase mu | P28827 | PTPRM | -0.34 | 1.84E-06 | 5.54E-06 | 0.20 | 1.50E-01 | 2.02E-01 |
| Pentraxin-related protein PTX3 | P26022 | PTX3 | 2.21 | 1.71E-21 | 1.44E-19 | 2.19 | 7.71E-15 | 2.36E-13 |
| Ras-related protein Rab-6A | P20340 | RAB6A | -0.34 | 1.57E-01 | 1.94E-01 | 0.09 | 7.57E-01 | 7.92E-01 |
| Rab GTPase-activating protein 1-like | Q5R372 | RABGAP1L | 0.65 | 5.56E-05 | 1.20E-04 | 0.77 | 5.76E-03 | 1.08E-02 |
| Regenerating islet-derived protein 4 | Q9BYZ8 | REG4 | -0.74 | 5.17E-09 | 2.32E-08 | 0.01 | 9.80E-01 | 9.80E-01 |
| Roundabout homolog 1 | Q9Y6N7 | ROBO1 | 0.30 | 3.31E-06 | 9.46E-06 | 0.77 | 6.25E-08 | 3.15E-07 |
| Sterile alpha motif domain-containing protein 9-like | Q8IVG5 | SAMD9L | 1.91 | 8.64E-10 | 4.55E-09 | 2.76 | 4.59E-11 | 5.53E-10 |
| Secretogranin-3 | Q8WXD2 | SCG3 | 0.99 | 6.08E-09 | 2.59E-08 | 1.16 | 3.22E-05 | 9.04E-05 |
| Uteroglobin | P11684 | SCGB1A1 | -0.12 | 3.30E-01 | 3.94E-01 | -0.11 | 7.07E-01 | 7.54E-01 |
| Secretoglobin family 3A member 2 | Q96PL1 | SCGB3A2 | 0.02 | 8.97E-01 | 9.25E-01 | 0.44 | 2.24E-01 | 2.93E-01 |
| Secretagogin | O76038 | SCGN | -0.34 | 4.55E-03 | 7.38E-03 | 0.09 | 7.49E-01 | 7.89E-01 |
| Secernin-1 | Q12765 | SCRN1 | 1.29 | 3.92E-07 | 1.27E-06 | 2.53 | 8.31E-08 | 3.89E-07 |
| P-selectin glycoprotein ligand 1 | Q14242 | SELPLG | 0.15 | 1.11E-02 | 1.74E-02 | 0.25 | 1.65E-02 | 2.70E-02 |
| Serpin B8 | P50452 | SERPINB8 | 1.44 | 3.19E-09 | 1.54E-08 | 2.11 | 2.46E-06 | 8.38E-06 |
| SH2 domain-containing protein 1A | O60880 | SH2D1A | 0.93 | 1.74E-03 | 2.96E-03 | 2.33 | 2.14E-06 | 7.45E-06 |
| Serine hydroxymethyltransferase | P34896 | SHMT1 | 2.62 | 2.78E-09 | 1.36E-08 | 3.25 | 7.11E-08 | 3.38E-07 |
| Sialoadhesin | Q9BZZ2 | SIGLEC1 | 1.81 | 2.40E-18 | 1.09E-16 | 2.71 | 6.95E-20 | 1.17E-17 |
| Sialic acid-binding Ig-like lectin 10 | Q96LC7 | SIGLEC10 | 1.12 | 1.63E-11 | 1.12E-10 | 1.57 | 7.46E-10 | 6.14E-09 |
| Signal-regulatory protein beta-1 | O00241 | SIRPB1 | 1.47 | 7.32E-18 | 2.24E-16 | 2.26 | 6.04E-17 | 2.91E-15 |
| Signaling threshold-regulating transmembrane adapter 1 | Q9Y3P8 | SIT1 | 1.49 | 1.19E-11 | 8.72E-11 | 1.69 | 1.66E-06 | 6.07E-06 |
| Src kinase-associated phosphoprotein 2 | O75563 | SKAP2 | -0.39 | 3.80E-01 | 4.35E-01 | -0.02 | 9.76E-01 | 9.79E-01 |
| Signaling lymphocytic activation molecule | Q13291 | SLAMF1 | 0.63 | 2.75E-05 | 6.34E-05 | 0.73 | 4.54E-03 | 8.79E-03 |
| SLAM family member 7 | Q9NQ25 | SLAMF7 | 1.44 | 3.73E-14 | 4.84E-13 | 2.06 | 2.44E-11 | 3.43E-10 |
| Zinc transporter ZIP5 | Q6ZMH5 | SLC39A5 | 0.25 | 9.51E-02 | 1.21E-01 | 0.85 | 8.43E-03 | 1.54E-02 |
| SPARC-related modular calcium-binding protein 2 | Q9H3U7 | SMOC2 | -0.84 | 1.43E-09 | 7.30E-09 | -0.39 | 4.93E-02 | 7.23E-02 |
| Acid sphingomyelinase-like phosphodiesterase 3a | Q92484 | SMPDL3A | 0.72 | 4.33E-09 | 2.00E-08 | 1.43 | 5.62E-06 | 1.86E-05 |
| Serine peptidase inhibitor | O60575 | SPINK4 | -0.30 | 1.17E-02 | 1.81E-02 | 0.40 | 1.83E-01 | 2.44E-01 |
| Kunitz-type protease inhibitor 2 | O43291 | SPINT2 | -0.05 | 5.67E-01 | 6.26E-01 | 0.15 | 4.14E-01 | 4.86E-01 |
| Spondin-1 | Q9HCB6 | SPON1 | 0.18 | 7.12E-02 | 9.30E-02 | 0.44 | 3.84E-02 | 5.80E-02 |
| Protein sprouty homolog 2 | O43597 | SPRY2 | -0.29 | 6.84E-02 | 8.97E-02 | -1.00 | 2.81E-03 | 5.71E-03 |
| SRSF protein kinase 2 | P78362 | SRPK2 | 1.37 | 6.64E-07 | 2.09E-06 | 1.39 | 1.27E-04 | 3.25E-04 |
| Syntaxin-8 | Q9UNK0 | STX8 | -0.04 | 8.51E-01 | 8.86E-01 | 0.41 | 9.35E-02 | 1.31E-01 |
| Bile salt sulfotransferase | Q06520 | SULT2A1 | 2.16 | 5.40E-09 | 2.36E-08 | 3.41 | 7.01E-09 | 4.45E-08 |
| TRAF family member-associated NF-kappa-B activator | Q92844 | TANK | 0.16 | 3.54E-01 | 4.13E-01 | 0.35 | 1.33E-01 | 1.82E-01 |
| TBC1 domain family member 5 | Q92609 | TBC1D5 | 0.24 | 3.52E-01 | 4.13E-01 | 0.83 | 2.21E-02 | 3.51E-02 |
| Trefoil factor 2 | Q03403 | TFF2 | -0.02 | 8.27E-01 | 8.70E-01 | 0.59 | 1.51E-02 | 2.51E-02 |
| Protransforming growth factor alpha | P01135 | TGFA | -0.22 | 1.55E-01 | 1.91E-01 | 0.23 | 3.91E-01 | 4.64E-01 |
| Transforming growth factor beta-1 | P01137 | TGFB1 | 0.41 | 1.97E-04 | 3.94E-04 | 0.80 | 1.66E-04 | 4.09E-04 |
| Metalloproteinase inhibitor 3 | P35625 | TIMP3 | 1.00 | 7.75E-06 | 1.91E-05 | 1.26 | 4.37E-05 | 1.20E-04 |
| Toll-like receptor 3 | O15455 | TLR3 | 0.93 | 1.74E-09 | 8.77E-09 | 1.07 | 4.27E-03 | 8.42E-03 |
| Tumor necrosis factor | P01375 | TNF | 1.35 | 5.74E-09 | 2.48E-08 | 1.90 | 7.91E-07 | 3.14E-06 |
| Tumor necrosis factor receptor superfamily member 11A; Receptor activator of nuclear factor-kappa Beta (RANK) | Q9Y6Q6 | TNFRSF11A | 0.25 | 5.25E-02 | 7.07E-02 | 0.94 | 1.08E-03 | 2.40E-03 |
| Tumor necrosis factor receptor superfamily member 11B; Osteoprotegerin (OPG) | O00300 | TNFRSF11B | 2.09 | 1.47E-13 | 1.67E-12 | 2.09 | 1.19E-07 | 5.44E-07 |
| Tumor necrosis factor receptor superfamily member 13B; Transmembrane activator and CAML interactor (TACI) | O14836 | TNFRSF13B | 1.84 | 1.48E-13 | 1.67E-12 | 2.46 | 3.05E-11 | 4.02E-10 |
| Tumor necrosis factor receptor superfamily member 13C;  B-cell activating factor receptor (BAFF-R) | Q96RJ3 | TNFRSF13C | 0.02 | 8.42E-01 | 8.81E-01 | 0.18 | 3.90E-01 | 4.64E-01 |
| Tumor necrosis factor receptor superfamily member 14; Herpesvirus entry mediator (HVEM) | Q92956 | TNFRSF14 | 0.60 | 6.59E-09 | 2.74E-08 | 1.25 | 1.84E-08 | 1.07E-07 |
| Tumor necrosis factor receptor superfamily member 4;  OX40 receptor | P43489 | TNFRSF4 | -0.33 | 2.38E-03 | 4.04E-03 | 0.29 | 1.92E-01 | 2.55E-01 |
| Tumor necrosis factor ligand superfamily member 10;  TNF-related apoptosis-inducing ligand (TRAIL) | P50591 | TNFSF10 | 0.87 | 8.32E-11 | 5.19E-10 | 0.93 | 3.99E-05 | 1.10E-04 |
| Tumor necrosis factor ligand superfamily member 11;  Receptor activator of nuclear factor kappa-B ligand (RANKL) | O14788 | TNFSF11 | -2.38 | 1.74E-15 | 2.67E-14 | -1.93 | 3.79E-10 | 3.37E-09 |
| Tumor necrosis factor ligand superfamily member 12;  TNF-related weak inducer of apoptosis (TWEAK) | O43508 | TNFSF12 | -0.43 | 2.25E-05 | 5.29E-05 | -0.40 | 1.50E-02 | 2.51E-02 |
| Tumor necrosis factor ligand superfamily member 13;  A proliferation-inducing ligand (APRIL) | O75888 | TNFSF13 | 0.56 | 2.18E-04 | 4.26E-04 | 0.63 | 1.98E-02 | 3.18E-02 |
| Tripeptidyl-peptidase 1 | O14773 | TPP1 | 1.90 | 2.61E-16 | 5.16E-15 | 2.42 | 2.92E-13 | 6.27E-12 |
| Tryptase alpha/beta-1 | Q15661 | TPSAB1 | -0.06 | 4.88E-01 | 5.48E-01 | 0.36 | 3.28E-01 | 4.00E-01 |
| TNF receptor-associated factor 2 | Q12933 | TRAF2 | 0.79 | 2.43E-05 | 5.68E-05 | 0.85 | 4.43E-03 | 8.68E-03 |
| Triggering receptor expressed on myeloid cells 2 | Q9NZC2 | TREM2 | 0.30 | 4.04E-02 | 5.56E-02 | 0.72 | 6.62E-03 | 1.23E-02 |
| E3 ubiquitin-protein ligase Tripartite motif-containing protein 21 | P19474 | TRIM21 | 4.41 | 2.75E-16 | 5.16E-15 | 5.03 | 4.45E-12 | 6.52E-11 |
| Tripartite motif-containing protein 5 | Q9C035 | TRIM5 | 1.05 | 3.59E-04 | 6.69E-04 | 1.20 | 1.87E-03 | 4.02E-03 |
| Vascular endothelial growth factor A | P15692 | VEGFA | 0.28 | 1.70E-02 | 2.49E-02 | 0.79 | 3.74E-03 | 7.41E-03 |
| Vascular endothelial growth factor D | O43915 | VEGFD | 0.57 | 4.06E-08 | 1.48E-07 | 0.71 | 1.39E-04 | 3.48E-04 |
| WAP, Kazal, immunoglobulin, Kunitz and NTR domain-containing protein 2 | Q8TEU8 | WFIKKN2 | 0.27 | 5.75E-03 | 9.22E-03 | 0.36 | 7.47E-02 | 1.07E-01 |
| Protein Wnt-9a | O14904 | WNT9A | -0.16 | 5.28E-02 | 7.09E-02 | -0.12 | 4.54E-01 | 5.28E-01 |
| YTH domain-containing family protein 3 | Q7Z739 | YTHDF3 | 0.61 | 3.55E-05 | 8.03E-05 | 0.35 | 2.55E-01 | 3.27E-01 |
