# Supplementary material for: Longitudinal proteomic profiling of the inflammatory response in dengue patients
Source: PLoS Negl Trop Dis. 2023 Jan 3;17(1):e0011041. doi: 10.1371/journal.pntd.0011041 (PMC9838874; doi:10.1371/journal.pntd.0011041)
Supplement: S2 Table — (DOCX) [file pntd.0011041.s002.docx]

**S2 Table. Shared differentially expressed proteins (DEPs) and previously identified transcripts (DETs)**

| **No** | **Protein** | **Associated Clinical Parameters** | **No** | **Protein** | **Associated Clinical Parameters** |
| --- | --- | --- | --- | --- | --- |
| *Shared DEPs/DETs in acute versus convalescent phase/controls* | | | | | |
| 1. | ADA | *WBC, SDC1* | 28. | IL32 | *-* |
| 2. | ADGRE2 | *SDC1* | 29. | IL4R | *-* |
| 3. | BCR | *-* | 30. | KYNU | *SDC1* |
| 4. | C1QA | *-* | 31. | LAMP3 | *WBC, SDC1* |
| 5. | CCL20 | *SDC1* | 32. | LAP3 | *SDC1* |
| 6. | CCL3 | *HCT* | 33. | LGALS9 | *-* |
| 7. | CCL4 | *Age, WBC* | 34. | LILRB4 | *WBC* |
| 8. | CD276 | *-* | 35. | LRRN1 | *-* |
| 9. | CD79B | *-* | 36. | LY9 | *HCT* |
| 10. | CLEC4A | *HCT* | 37. | MZB1 | *Sec. dengue* |
| 11. | CRELD2 | *SDC1* | 38. | NBN | *-* |
| 12. | CXCL10 | *-* | 39. | NELL2 | *SDC1* |
| 13. | CXCL8 | *WBC, SDC1* | 40. | PIK3AP1 | *SDC1* |
| 14. | EPHA1 | *-* | 41. | PLAUR | *Age* |
| 15. | FCRL2 | *-* | 42. | PNPT1 | *WBC, SDC1* |
| 16. | FOXO1 | *Sec. dengue, WBC, SDC1* | 43. | SAMD9L | *-* |
| 17. | GALNT3 | *WBC* | 44. | SH2D1A | *Sec. dengue, WBC, SDC1* |
| 18. | GZMA | *GBWT* | 45. | SIGLEC1 | *WBC, SDC1* |
| 19. | GZMB | *GBWT* | 46. | SIGLEC10 | *Albumin* |
| 20. | HSPA1A | *ALT, SDC1* | 47. | SLAMF7 | *-* |
| 21. | IFNG | *WBC, SDC1* | 48. | SMPDL3A | *-* |
| 22. | IKBKG | *-* | 49 | TNF | *Age, GBWT* |
| 23. | IL10RA | *-* | 50. | TNFSF10 | *WBC* |
| 24. | IL15 | *WBC, SDC1* | 51. | TRIM21 | *WBC, SDC1* |
| 25. | IL15RA | *-* | 52. | TRIM5 | *-* |
| 26. | IL1R2 | *-* | 53 | VEGFA | *-* |
| 27. | IL1RN | *Age* |  |  |  |
| *Shared DEPs/DETs in severe versus non-severe dengue or dengue fever vs hemorrhagic fever* | | | | | |
| 1. | BACH1 | *SDC1* |  |  |  |
| 2. | CD244 | *PLT* |  |  |  |
| 3. | CXCL10 | *-* |  |  |  |
| 4. | CXCL8 | *WBC, SDC1* |  |  |  |
| 5. | IL10 | *WBC, SDC1* |  |  |  |
| 6. | IL15 | *WBC, SDC1* |  |  |  |
| 7. | IL17C | *ALT* |  |  |  |
| 8. | IL18 | *-* |  |  |  |
| 9. | IL1R2 | *-* |  |  |  |
| 10. | IL1RN | *Age* |  |  |  |
| 11. | KLRD1 | *ALT, SDC1* |  |  |  |
| 12. | LAMP3 | *WBC, SDC1* |  |  |  |
| 13. | NCR1 | *-* |  |  |  |
| 14. | SERPINB8 | *-* |  |  |  |
| 15. | TRAF2 | *-* |  |  |  |

HCT, hematocrit; WBC, white blood cell number; PLT, platelet number; ALT, alanine transferase; SDC1, plasma syndecan-1; Secondary dengue; probable secondary dengue infection, GBWT, gall-bladder wall thickness.
