# Supplementary material for: Longitudinal proteomic profiling of the inflammatory response in dengue patients
Source: PLoS Negl Trop Dis. 2023 Jan 3;17(1):e0011041. doi: 10.1371/journal.pntd.0011041 (PMC9838874; doi:10.1371/journal.pntd.0011041)
Supplement: S3 Table — (DOCX) [file pntd.0011041.s003.docx]

**S3 Table. Top 15 Gene Ontology Biological Process functional enrichments of the 53 shared DEPs/DETs**

| **No** | **GO Term** | **Strength** | **FDR** | **GENEs** |
| --- | --- | --- | --- | --- |
| 1 | Positive regulation of CD4+, CD25+, alpha-beta regulatory T cell differentiation involved in immune response | 2.57 | 0.003 | IFNG, LGALS9 |
| 2 | Positive regulation of Calcidiol 1-monooxygenase activity | 2.39 | 0.0046 | IFNG, TNF |
| 3 | Negative regulation of interleukin-1-mediated signaling pathway | 2.17 | 0.0079 | IL1RN, IL1R2 |
| 4 | Positive regulation of natural killer cell chemotaxis | 2.09 | 0.01 | CCL3, CCL4 |
| 5 | Negative regulation of amyloid-beta clearance | 1.97 | 0.0151 | IFNG, TNF |
| 6 | Positive regulation of regulatory t cell differentiation | 1.9 | 0.0011 | IFNG, LILRB4, LGALS9 |
| 7 | Negative regulation of myeloid leukocyte mediated immunity | 1.87 | 0.0207 | BCR, LGALS9 |
| 8 | Positive regulation of viral entry into host cell | 1.87 | 0.0207 | TRIM21, LGALS9 |
| 9 | Nucleoside triphosphate catabolic process | 1.79 | 0.0263 | SMPDL3A, ADA |
| 10 | Negative regulation of leukocyte degranulation | 1.79 | 0.0263 | BCR, LGALS9 |
| 11 | interleukin-15-mediated signaling pathway | 1.75 | 0.0295 | IL15, IL15RA |
| 12 | Astrocyte activation | 1.74 | 0.0024 | IFNG, C1QA, TNF |
| 13 | Negative regulation by host of viral transcription | 1.72 | 0.0329 | CCL3, CCL4 |
| 14 | T cell chemotaxis | 1.69 | 0.036 | CXCL10, CCL3 |
| 15 | Induction of positive chemotaxis | 1.69 | 0.036 | CXCL8, VEGFA |
