# Supplementary material for: Longitudinal proteomic profiling of the inflammatory response in dengue patients
Source: PLoS Negl Trop Dis. 2023 Jan 3;17(1):e0011041. doi: 10.1371/journal.pntd.0011041 (PMC9838874; doi:10.1371/journal.pntd.0011041)
Supplement: S1 Fig — (DOCX) [file pntd.0011041.s004.docx]

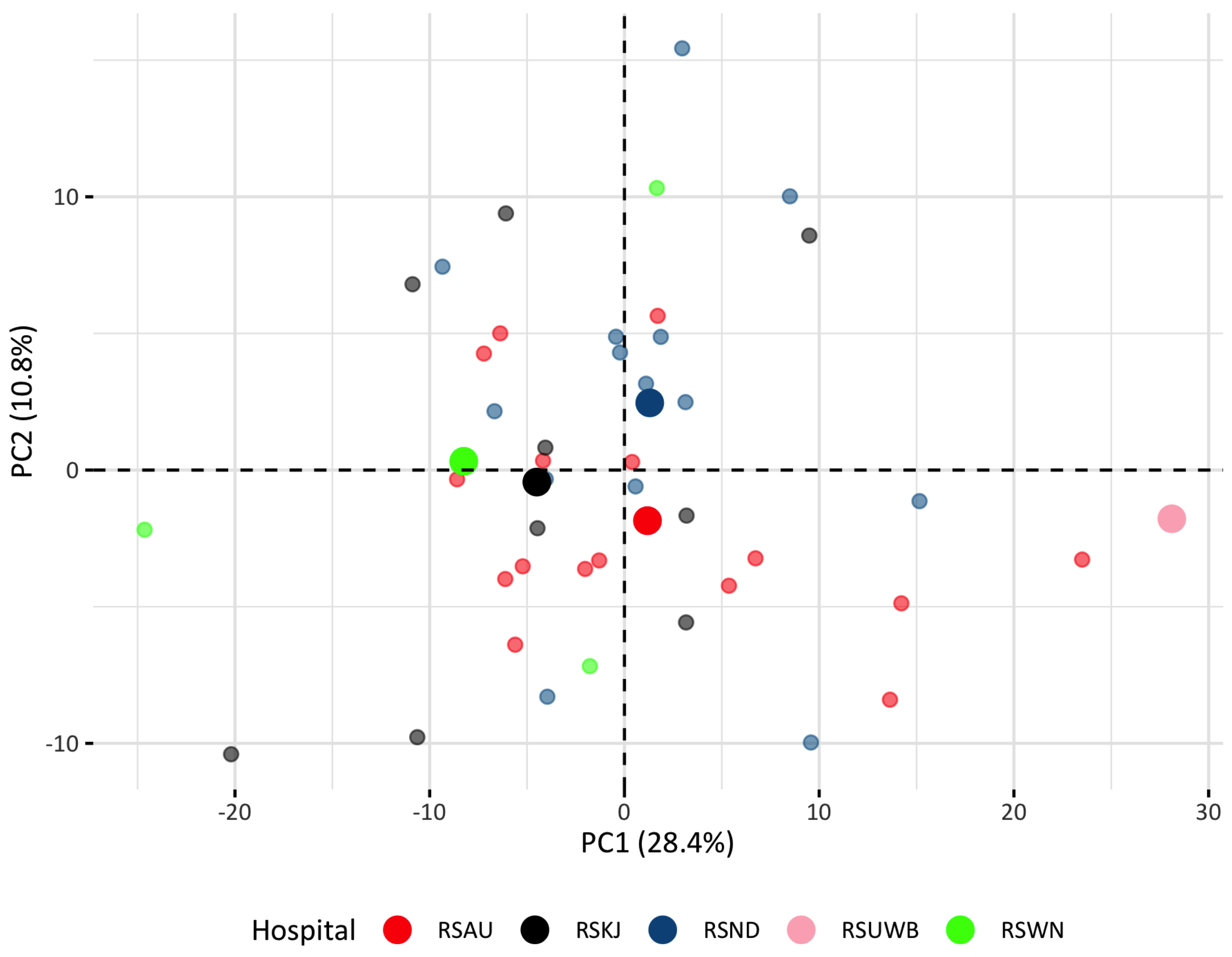


**S1 Fig. Principal component analysis of individual acute dengue patients across different hospitals.**  RSAU = Dr. M. Salamun Air Force in Bandung; RSKJ = Kartini General Hospital in Jepara; RSND = Diponegoro National University Hospital; RSUWB = William Booth General Hospital; RSWN= Wongsonegoro General Hospital in Semarang. Larger dots represent the central of cluster.
