# Supplementary material for: Longitudinal proteomic profiling of the inflammatory response in dengue patients
Source: PLoS Negl Trop Dis. 2023 Jan 3;17(1):e0011041. doi: 10.1371/journal.pntd.0011041 (PMC9838874; doi:10.1371/journal.pntd.0011041)
Supplement: S2 Fig — (DOCX) [file pntd.0011041.s005.docx]

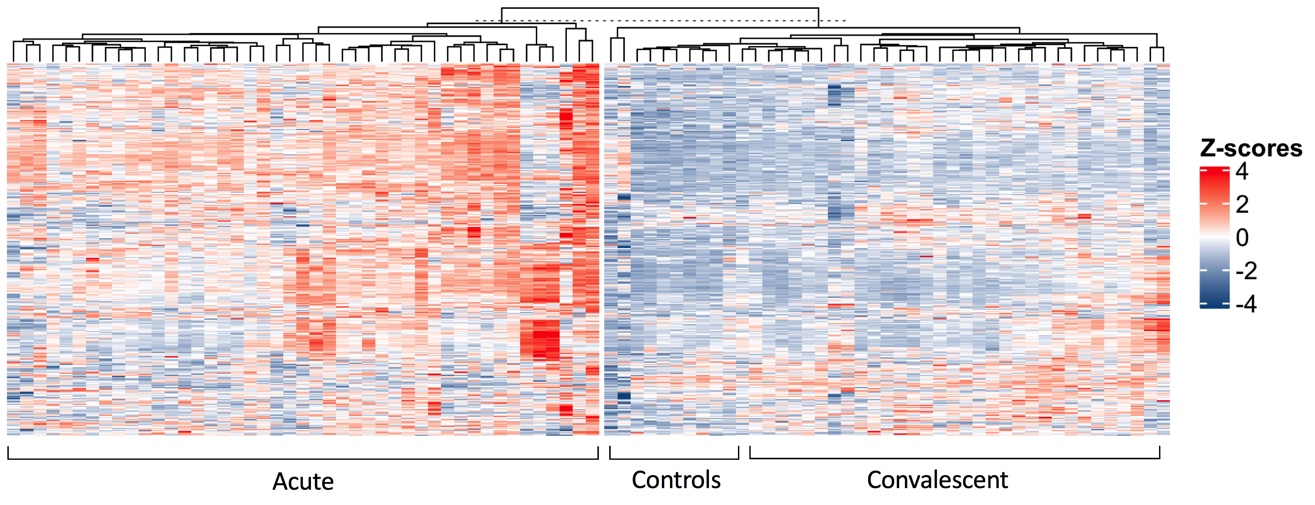


**S2 Fig.** **Hierarchical clustering analysis.** Depicted a heatmap of an unsupervised hierarchical clustering analysis of the 337 inflammatory proteins from dengue patients in the acute phase (N=43), convalescent phase (N=35) and controls (N=10).
