# Supplementary material for: Longitudinal proteomic profiling of the inflammatory response in dengue patients
Source: PLoS Negl Trop Dis. 2023 Jan 3;17(1):e0011041. doi: 10.1371/journal.pntd.0011041 (PMC9838874; doi:10.1371/journal.pntd.0011041)
Supplement: S3 Fig — (DOCX) [file pntd.0011041.s006.docx]

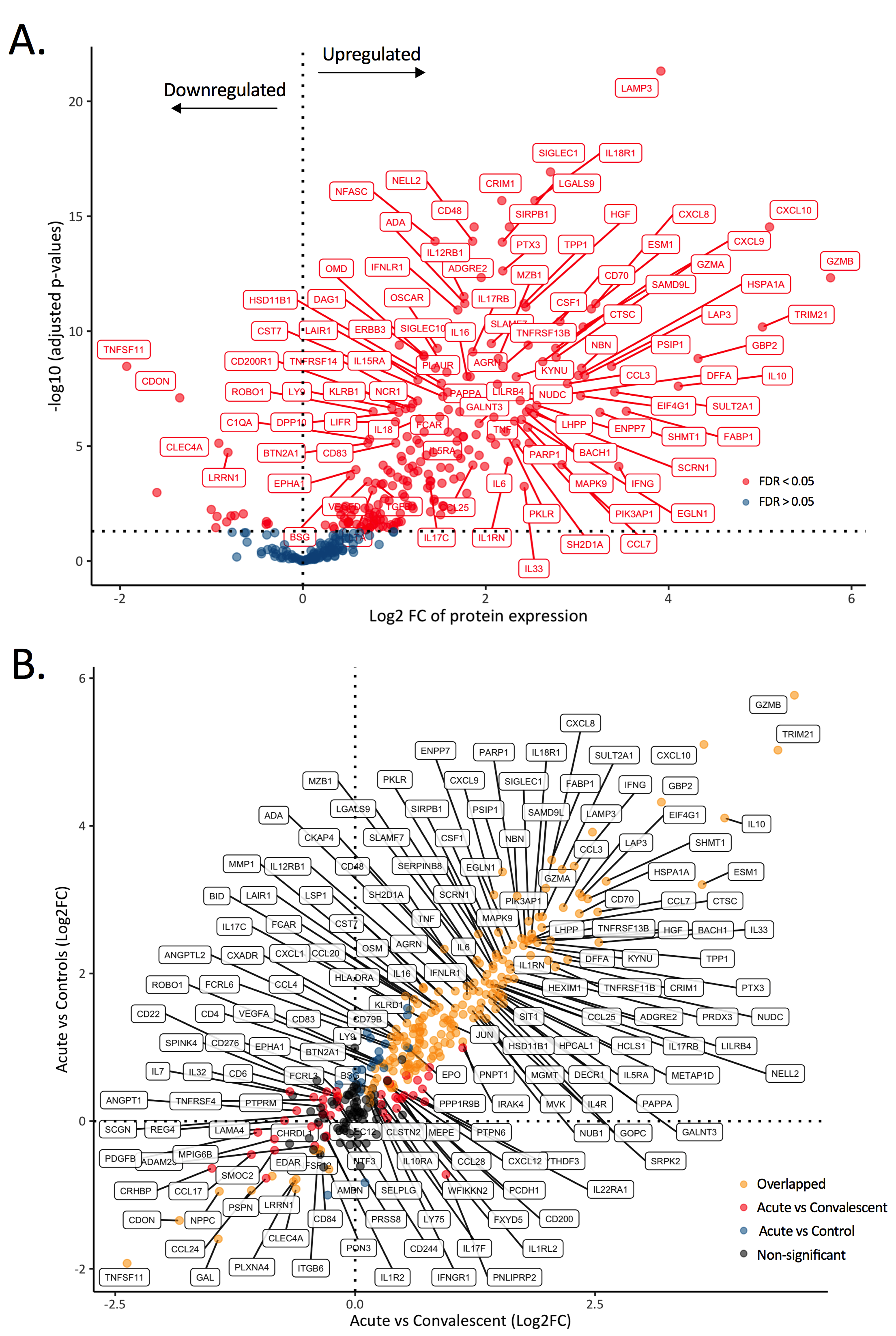


**S3 Fig. Overlapped differentially expressed proteins (DEPs) between acute vs convalescent dengue and acute dengue vs healthy controls.** (A). A volcano plot displaying the DEPs between acute phase of dengue patients (N = 43) versus adult healthy controls (N = 10). Depicted X-axis Log2 Fold-Change (Log2 FC) of protein expression (Acute versus Controls) and Y-axis (-Log10) of adjusted *p*-value (Benjamini-Hochberg false-discovery rate). (B). A scatterplot quadrant analysis of the differentially overlapped proteins between Acute vs Convalescent and Acute vs Control samples.
