# Supplementary material for: Longitudinal proteomic profiling of the inflammatory response in dengue patients
Source: PLoS Negl Trop Dis. 2023 Jan 3;17(1):e0011041. doi: 10.1371/journal.pntd.0011041 (PMC9838874; doi:10.1371/journal.pntd.0011041)
Supplement: S4 Fig — (DOCX) [file pntd.0011041.s007.docx]

**
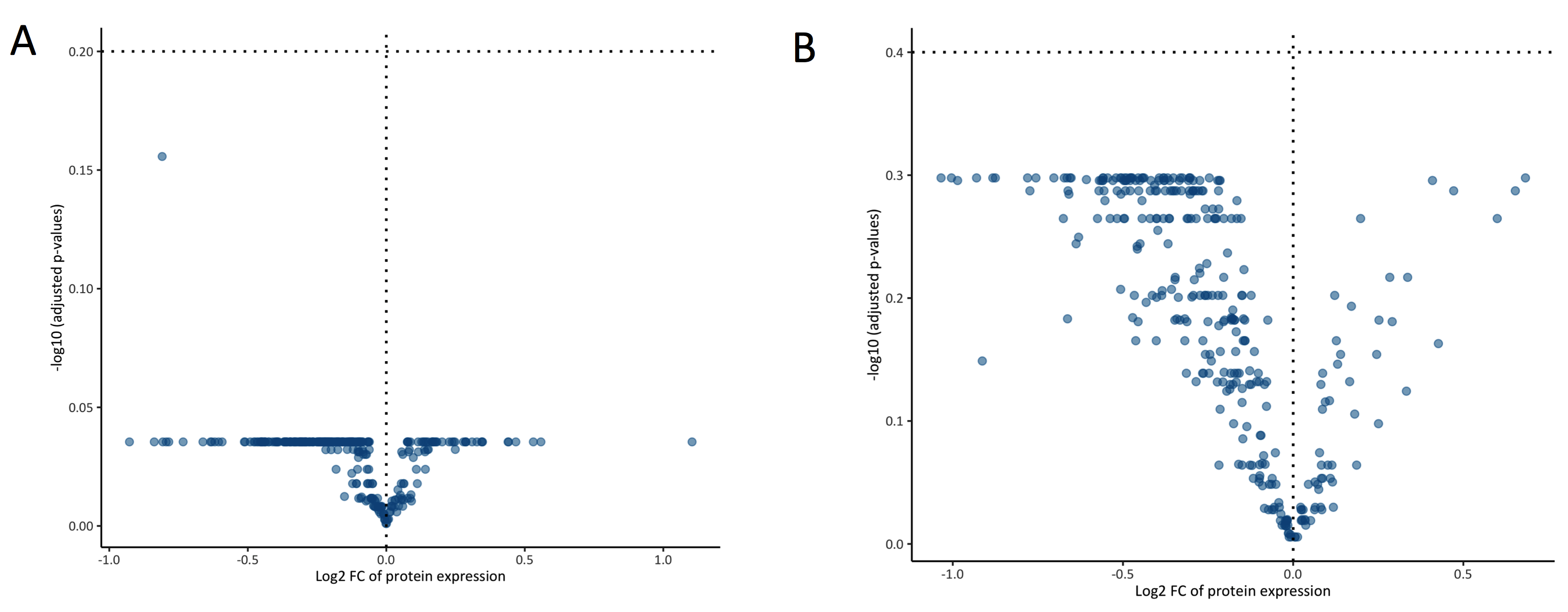
**

**S4 Fig. Differentially expressed proteins (DEPs) between dengue hemorrhagic fever (DHF) vs dengue fever (DF) patients. (A)**. A volcano plot displaying the DEPs between acute phase of DHF patients (N = 18) versus DF patients (N = 25) at enrollment. **(B)**. A volcano plot displaying the DEPs between acute phase of DHF patients (N = 26) versus DF patients (N = 17) during hospitalization. Depicted X-axis Log2 Fold-Change (Log2 FC) of protein expression (DHF versus DF) and Y-axis (-Log10) of adjusted *p*-value (Benjamini-Hochberg false-discovery rate).
