# Supplementary material for: Longitudinal proteomic profiling of the inflammatory response in dengue patients
Source: PLoS Negl Trop Dis. 2023 Jan 3;17(1):e0011041. doi: 10.1371/journal.pntd.0011041 (PMC9838874; doi:10.1371/journal.pntd.0011041)
Supplement: S5 Fig — (DOCX) [file pntd.0011041.s008.docx]

**
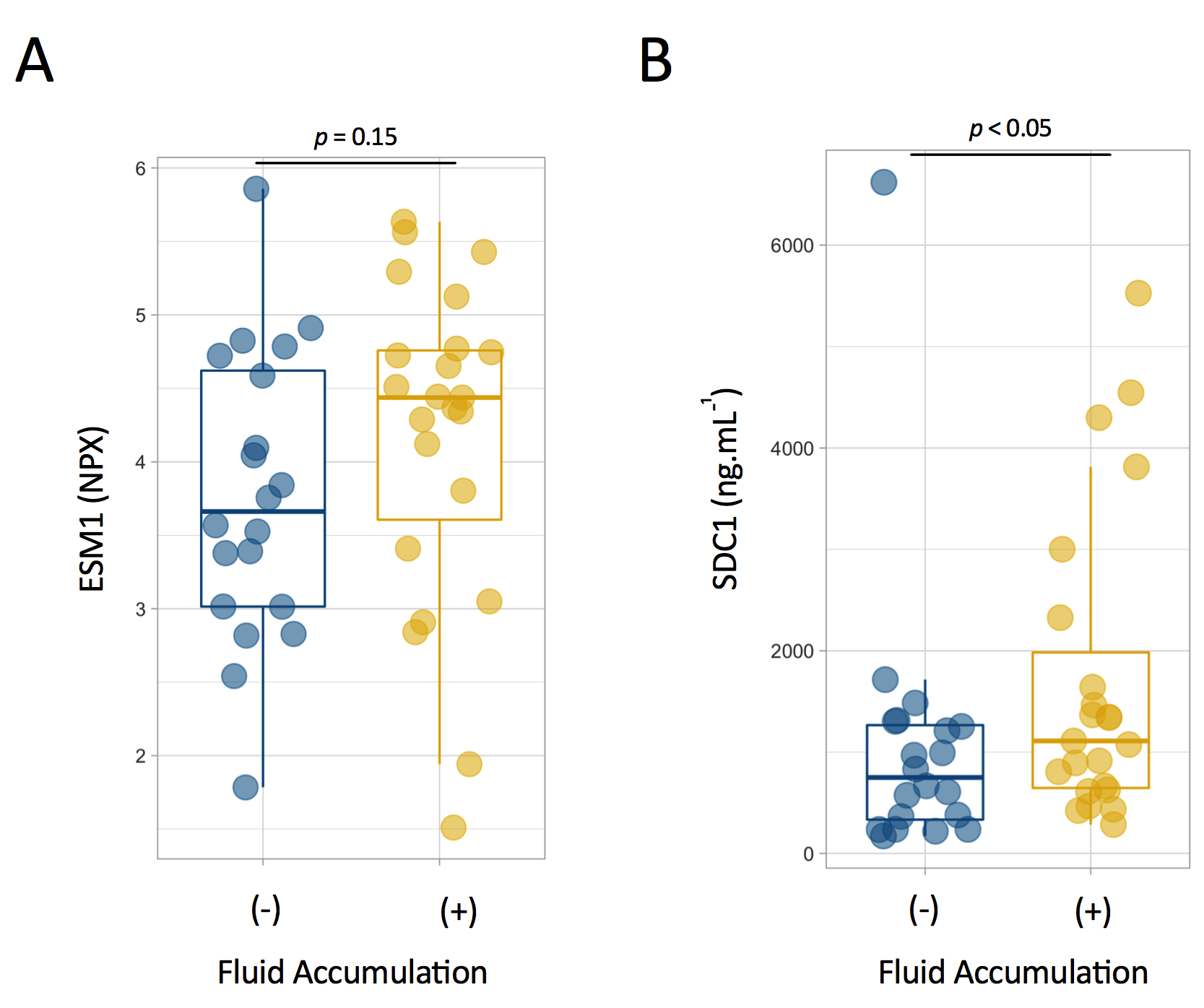
**

**S5 Fig.** **Circulating Endothelial Cell Specific Molecule 1 (ESM1) and Syndecan-1 (SDC1) on acute phase in the presence of fluid accumulation during hospitalization**. **(A)** Plasma normalized protein expression (NPX) of ESM1 and **(B)** plasma concentration (ng. mL^-1^) of SDC1 in the acute phase grouped based on the presence of fluid accumulation during hospitalization. Depicted are individual data (N=43) together with a box plot showing median with interquartile range. Differential expression was analyzed using the Mann-Whitney U test. Fluid accumulation was defined as the presence of ascites and/or pleural fluid as determined by a serial bedside abdominal ultrasonography.
