# Supplementary material for: Longitudinal proteomic profiling of the inflammatory response in dengue patients
Source: PLoS Negl Trop Dis. 2023 Jan 3;17(1):e0011041. doi: 10.1371/journal.pntd.0011041 (PMC9838874; doi:10.1371/journal.pntd.0011041)
Supplement: S6 Fig — (DOCX) [file pntd.0011041.s009.docx]

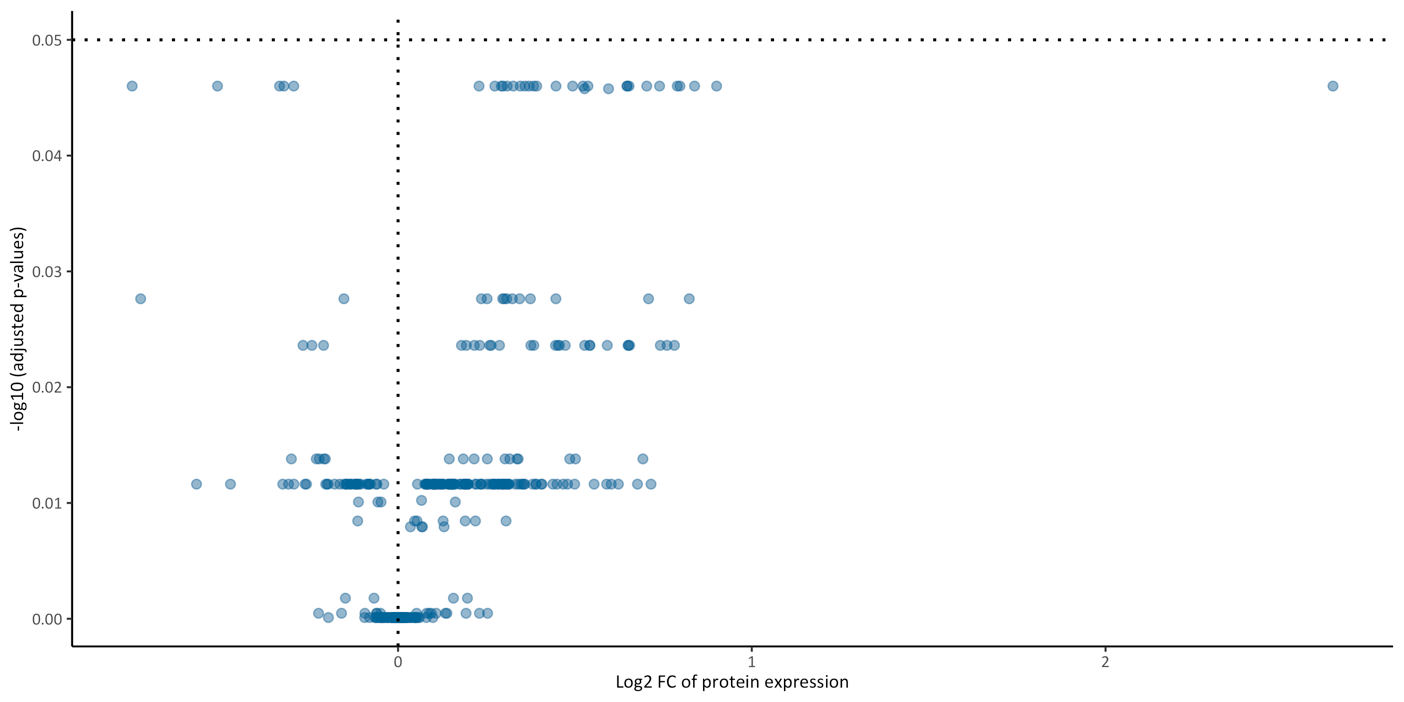


**S6 Fig. Differentially expressed proteins (DEPs) in convalescent phase between Oseltamivir arm versus Placebo arm.** A volcano plot displaying the DEPs between convalescent phase of dengue patients receiving Oseltamivir (N = 10) versus placebo (N = 25). Depicted X-axis Log2 Fold-Change (Log2 FC) of protein expression (Oseltamivir versus Placebo) and Y-axis (-Log10) of adjusted p-value (Benjamini-Hochberg false-discovery rate).
