# Supplementary material for: Longitudinal proteomic profiling of the inflammatory response in dengue patients
Source: PLoS Negl Trop Dis. 2023 Jan 3;17(1):e0011041. doi: 10.1371/journal.pntd.0011041 (PMC9838874; doi:10.1371/journal.pntd.0011041)
Supplement: S7 Fig — (DOCX) [file pntd.0011041.s010.docx]

**
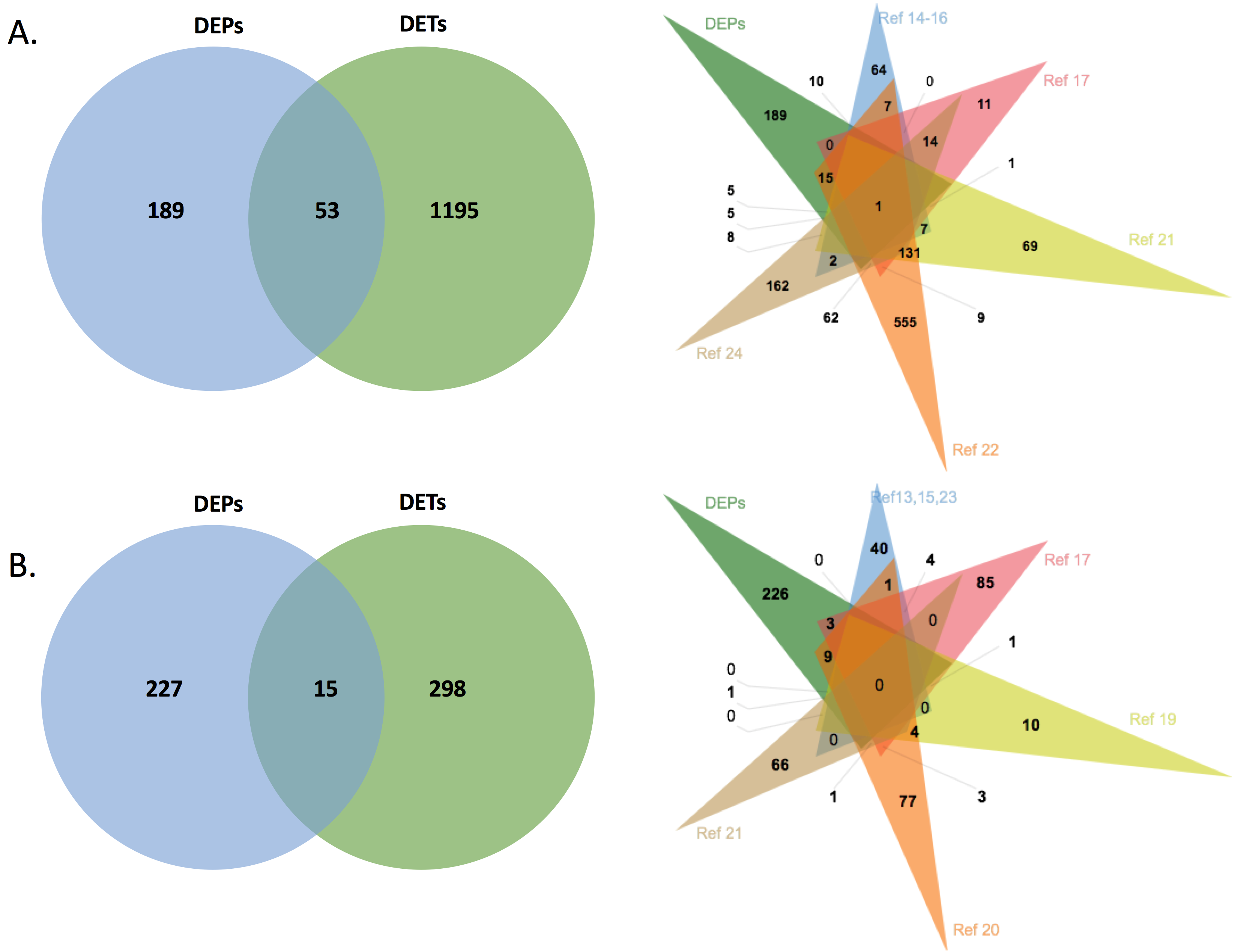
**

**S7 Fig. Shared differentially expressed proteins (DEPs) and previously identified transcripts (DETs) from dengue patients.** DEPs identified in this study were intersected with DETs identified in previous studies. **(A)** Venn chart for the number of shared DEPs/DETs in acute versus convalescent phase or control samples, as well as shared DEPs/DETs per cohort. **(B)** Venn chart for the number of shared DEPs/DETs in severe versus non-severe dengue, as well as shared DEPs/DETs per cohort. Ref: [14-16] as well as Ref: [13,15,23] were combined due to each total genes < 120 genes.
