# Supplementary material for: Longitudinal proteomic profiling of the inflammatory response in dengue patients
Source: PLoS Negl Trop Dis. 2023 Jan 3;17(1):e0011041. doi: 10.1371/journal.pntd.0011041 (PMC9838874; doi:10.1371/journal.pntd.0011041)
Supplement: Attachment — Submitted filename: PNTD_ Einav review_Aug 7.docx [file pntd.0011041.s011.docx]

The manuscript by Garishah et al. aims at improving the understanding of the changes in inflammatory pathways and the pattern of host defense responses during acute dengue virus (DENV) infection. Based on a Proximity Extension Analysis (PEA) applied to longitudinal patient-derived plasma samples, the authors report an unbiased proteomic characterization wherein over 200 inflammatory proteins were found to be upregulated in the acute phase of dengue infection relative to the convalescent phase.

The novelty of the study lies in the associations observed between interferon-mediated pathways and other inflammatory proteins to circulating concentrations of syndecan-1, a surrogate marker for endothelial glycocalyx disruption. While it is hypothesized that vascular complications during DENV infection are directly affected by disruption of the endothelial glycocalyx, the inflammatory pathways involved in this process are to date not fully understood. The use of the PEA technology is also quite novel, and it confers an enhanced specificity and sensitivity than other techniques like mass spectrometry, with the advantage that a high number of inflammatory proteins can be analyzed simultaneously.

While a major limitation of the study is that it does not include patients who progressed to severe dengue, it does contribute to the understanding of the host immune responses to uncomplicated dengue infection. Moreover, it offers an opportunity for the discovery and validation of new disease biomarkers.

The manuscript is generally well-written. Some comments that the authors should address are listed below:

**Comment 1:** It is well established that antibody-dependent enhancement and secondary infections play a major role in the pathogenesis of dengue infection. Ideally, it would be important to correlate the expression level of the identified differentially expressed proteins with prior dengue exposure (primary vs. secondary infection).

**Comment 2:**  High viral loads have been reported in some studies to be associated with disease severity. It would be interesting to assess and correlate viral loads and the differentially expressed proteins, together with the clinical parameters described in the study.

**Comment 3:** This study was performed with a cohort of patients participating in a randomized trial evaluating the effect of oseltamivir on platelet recovery and plasma leakage in adults with dengue and thrombocytopenia. While acute samples were taken prior to drug administration, convalescent samples were taken afterwards. Potential secondary effects of the drug should therefore be considered and discussed as a limitation of the study.

**Comment 4:** The genetic background of the control subjects included in this study (from the Netherlands) was different from that of the patients (Indonesia). This should be mentioned as a limitation in the discussion.
