# Supplementary material for: Longitudinal proteomic profiling of the inflammatory response in dengue patients
Source: PLoS Negl Trop Dis. 2023 Jan 3;17(1):e0011041. doi: 10.1371/journal.pntd.0011041 (PMC9838874; doi:10.1371/journal.pntd.0011041)
Supplement: Attachment — Submitted filename: Point to Point Response.docx [file pntd.0011041.s012.docx]

**Point-to-Point Response**

*(For line numbering, please refer to our clean manuscript.docx)*

**Reviewer Comments**

**Summary and General Comments**

**Reviewer #1**:

The manuscript by Garishah et al. aims at improving the understanding of the changes in inflammatory pathways and the pattern of host defense responses during acute dengue virus (DENV) infection. Based on a Proximity Extension Analysis (PEA) applied to longitudinal patient-derived plasma samples, the authors report an unbiased proteomic characterization wherein over 200 inflammatory proteins were found to be upregulated in the acute phase of dengue infection relative to the convalescent phase.

The novelty of the study lies in the associations observed between interferon-mediated pathways and other inflammatory proteins to circulating concentrations of syndecan-1, a surrogate marker for endothelial glycocalyx disruption. While it is hypothesized that vascular complications during DENV infection are directly affected by disruption of the endothelial glycocalyx, the inflammatory pathways involved in this process are to date not fully understood. The use of the PEA technology is also quite novel, and it confers an enhanced specificity and sensitivity than other techniques like mass spectrometry, with the advantage that a high number of inflammatory proteins can be analyzed simultaneously.

While a major limitation of the study is that it does not include patients, who progressed to severe dengue, it does contribute to the understanding of the host immune responses to uncomplicated dengue infection. Moreover, it offers an opportunity for the discovery and validation of new disease biomarkers.

The manuscript is generally well-written. Some comments that the authors should address are listed below:

**Comment 1:** It is well established that antibody-dependent enhancement and secondary infections play a major role in the pathogenesis of dengue infection. Ideally, it would be important to correlate the expression level of the identified differentially expressed proteins with prior dengue exposure (primary vs. secondary infection).

**Our response:** We thank the reviewer for this suggestion. In response to the comment, we have categorized the enrolled patients in presumed primary infection (positive NS1 or IgM anti-DENV with negative IgG anti-DENV) or presumed secondary infection (positive IgG anti-DENV) **(revised Table 1)**. We have included secondary dengue as a parameter in **revised** **Figure 4**. Our analyses show that participants classified as having a secondary dengue infection had higher concentrations of different molecules involved in the host immune response, including lymphocyte function (CEACAM21, FOXO1, MZB1, PARP1, PPP1R9B, PSIP1, SH2D1A), glucocorticoid hormone regulation (CRHBP) and megakaryocyte maturation (MPIG6B). In contrast, no differences were observed in concentrations of pro-inflammatory cytokines, interferon-inducible effector proteins or granzymes.

We have changed the following in the manuscript:

a) We have included how participants were classified into presumed primary or secondary dengue in the methods “*Patients with a positive NS1 or IgM anti-DENV and negative IgG anti-DENV during hospitalization were considered as probable primary dengue infection, the remainder were considered as probable secondary dengue infection.*” **(Line 121-123)**.
b) We have included the number of presumed primary or secondary infections in the text of the results and in the revised **Table 1**: “*The dengue patients were enrolled at a median of 5 days (IQR, 4-5) after symptom onset; 25 (58.1%) patients were classified as probable primary dengue infection and 18 (41.9%) as probable secondary infection.”* **(Line 183-185)**.
c) We have included ‘probable secondary dengue infection’ as a variable in **revised** **Figure 4** and included the following text in the result section “*Participants classified as having a probable secondary dengue infection had higher concentrations of different molecules involved in the host immune response, including lymphocyte function (CEACAM21, FOXO1, MZB1, PARP1, PPP1R9B, PSIP1, SH2D1A), glucocorticoid hormone regulation (CRHBP) and megakaryocyte maturation (MPIG6B). In contrast, no differences were observed in concentrations of pro-inflammatory cytokines, interferon-inducible effector proteins or granzymes.”* **(Line 310-315).**

**Comment 2:** High viral loads have been reported in some studies to be associated with disease severity. It would be interesting to assess and correlate viral loads and the differentially expressed proteins, together with the clinical parameters described in the study.

**Response:** Unfortunately, viral load was not measured in the trial and it was also not possible to measure this in retrospect. Viremia is also known to peak early during the febrile phase and declines thereafter. Our study enrolled hospitalized patients with a median day of illness of 5 days and it is likely that viral loads had already declined markedly in most of these participants. We have included the fact that viral loads are not available as a limitation in the discussion section “*Second, high viral loads have been reported to be associated with disease severity in some studies, but viral loads were unavailable in our study.”* **(Line 418-419)**.

**Comment 3:** This study was performed with a cohort of patients participating in a randomized trial evaluating the effect of oseltamivir on platelet recovery and plasma leakage in adults with dengue and thrombocytopenia. While acute samples were taken prior to drug administration, convalescent samples were taken afterwards. Potential secondary effects of the drug should therefore be considered and discussed as a limitation of the study.

**Response:** We consider it is unlikely that oseltamivir, which was given for a maximum of five days, would still have effects on the plasma proteome in the convalescence phase 3-4 weeks later. This notion is supported by the fact that differential expression (DE) analysis of proteins in the convalescence phase did not reveal significant differences between samples from the oseltamivir or placebo arm. We have included this information in the results and included a Supplementary figure showing the DEP analysis: *“Ten convalescent samples were from participants allocated to the oseltamivir group, but there were no significant differences in protein concentrations in convalescent plasma from those allocated to the oseltamivir or placebo group* ***(S6 Fig)****.”* **(Line 204-207)**. In addition, we included this information as a limitation in the discussion “*Third, ten convalescence samples were from participants allocated to the oseltamivir group. Even though we cannot rule out potential secondary effects of the drug, there were at least two weeks between the last dose of oseltamivir and DEP analysis did not reveal significant differences between samples from participants allocated to the oseltamivir or placebo group.*” **(Line 420-423)**.

**S6 Fig. Differentially expressed proteins (DEPs) in convalescent phase between Oseltamivir arm versus Placebo arm.** A volcano plot displaying the DEPs between convalescent phase of dengue patients receiving Oseltamivir (N = 10) versus placebo (N = 25). Depicted X-axis Log2 Fold-Change (Log2 FC) of protein expression (Oseltamivir versus Placebo) and Y-axis (-Log10) of adjusted p-value (Benjamini-Hochberg false-discovery rate).

**Comment 4:** The genetic background of the control subjects included in this study (from the Netherlands) was different from that of the patients (Indonesia). This should be mentioned as a limitation in the discussion.

**Response:** The controls were actually adult Indonesians who were temporarily living in the Netherlands. We have clarified this in the methods **(Line 126-128)** and results **(Line 182-183)**.

**Reviewer #2:**

This study by Garishah and colleagues investigates differentially-regulated proteins within the plasma of convalescent and severe Dengue patients in a multicenter longitudinal cohort, using a proprietary proteomics technology (Olink). The authors identify a number of up- and down-regulated proteins, presenting a STRING network analysis and correlation analysis of some of these proteins with known clinical profiles.

The study is well-written, the clinical data collection and the composition of the cohorts well-designed and the proteomics data are presented clearly and concisely. However, the study at this stage seems rather preliminary as it mostly present one descriptive dataset without any follow-ups (see details below).

**Response:** We thank reviewer 2 for the evaluation and the comments. Regarding the comment that our study is rather preliminary, we refer to our response to the comments on the methods below.

--------------------

**Reviewer's Responses to Questions**

Key Review Criteria Required for Acceptance?

As you describe the new analyses required for acceptance, please consider the following:

**Methods**

-Are the objectives of the study clearly articulated with a clear testable hypothesis stated?

-Is the study design appropriate to address the stated objectives?

-Is the population clearly described and appropriate for the hypothesis being tested?

-Is the sample size sufficient to ensure adequate power to address the hypothesis being tested?

-Were correct statistical analysis used to support conclusions?

-Are there concerns about ethical or regulatory requirements being met?

**Reviewer #1:** I am not a statistician, however, based on what I am seeing, the sample size seems adequate to me and the statistical analysis supports the data. No ethical concerns.

**Response:** N/A

**Reviewer #2:** Major points:

The study holds some potential since the patient cohorts is well defined, longitudinal and multicenter. The data appear adequately analyzed (including multiple hypothesis testing and some modelling; see i.e. LIMMA); however, in its current stage the study appears rather preliminary and even the bioinformatic analysis is rather superficial in scope for a descriptive study, thereby limiting its relevance for both clinicians or virologists.

The authors present the outcome of a commercially-provided service (Olink technology), identifying a number of expected and unexpected differentially regulated proteins in the plasma (i.e. PPP) of both convalescent and severe patients. While this approach has certain advantages highlighted by the authors, it does not represent a novelty or an incremental benefit over other plasma proteomics methods, which have currently reached 3X the depth of the method used (see ie. https://www.embopress.org/doi/full/10.15252/emmm.201910427). Nonetheless, the identified proteins reported herein might be of great interest or relevance if they would be validated in downstream experiments either in vitro or in silico.

For some of the proteins robustly up- or down-regulated and not directly involved in cytokine responses (i.e. GBP2, HEXIM1, LAP3, etc..), it would be interesting and important to assess their relevance in vitro (i.e. upon overexpression/silencing in monocytes or macrophages) or confirm their up- or down-regulations by alternative approaches in clinical samples. Similarly, it would be interesting to follow up on 1 or 2 candidate proteins exhibiting high correlation with certain clinical parameters (i.e. HGF or IL18R1, Fig 4).

Alternatively, if the authors aim to deliver a resource paper for the community, it would be important to provide thorough intersection of these results with all or most of the other published studies on plasma proteins profiling of DENV patients.

**Response:**

We thank the reviewer for these suggestions. Our study should indeed be seen as an explorative, hypothesis-generating study. Regarding the comparison with other proteomics technologies: the PEA technology used in our study is a highly sensitive, targeted immunoassay that has shown its value in understanding the biology of a rapidly increasing number of conditions. Studies have compared this targeted platform with untargeted (mass spectrometry; LC-MS/MS) plasma proteomics platforms highlighting the limitations and the advantages of both technologies and their complementary strengths (1).

Regarding the suggestion to perform validations: a large number of differential proteins were identified. We hope for your understanding that *in vitro* validation of their effects on inflammation and/or endothelial permeability is beyond the scope of this hypothesis-generating study. Moreover, previous *in vivo* and *in vitro* studies have already demonstrated the importance of quite some of the proteins identified in our study in the biology of dengue. We have included references to these previous studies in **Table 2** of our manuscript.

We thank the reviewer for the suggestion to intersect our results with the results from previous *omics* studies as an alternative approach for validating the results. Whereas multiple studies have provided blood transcriptome data in dengue, only three studies have used mass spectrometry (2-4). None of the differentially expressed proteins (DEPs) identified in our study overlapped with earlier reported proteins in the mass spectrometry studies. In contrast, intersection of the 242 DEPs identified in our study with 1248 differentially expressed transcripts (DETs) identified in studies comparing gene profiles between acute and convalescent dengue samples (5-16), yielded 53 (21.9%) shared DEPs/DETs **(S7 Fig A** and **S2 table)**, of which 38 (73%) in the same direction **(Figure 1D).** Most of the shared DEPs/DETs correlated with syndecan-1 (SDC1) concentrations or clinical parameters **(S2 Table).** According to the gene ontology database, the shared DEPs/DETs were mainly involved in lymphocyte chemotaxis (CXCL10, CCL3, CCL4), interleukin-15 mediated signaling (IL15, IL15RA) and negative regulation of host viral transcription (TRIM21, LGALS9, CCL3, CCL4) (**S3 Table**). Intersecting the DEPs with DETs associated with severe versus non-severe dengue or dengue fever versus dengue hemorrhagic fever (n=313) yielded 15 (6.2%) shared DEPs/DETs (**S7 Fig B and S2 table).**

We have changed the following in the manuscript:

a) We have now included the validation analysis above in the methods section “*To further validate our findings, we intersected the DEPs identified in our study with differentially expressed transcripts (DETs) reported in earlier studies that compared gene profiles between acute versus convalescent dengue patients or controls or between patients with severe versus non-severe illness [13-24].*” **(Line 174-177).**
b) We revised **Figure 1** by adding an additional panel D that shows the direction of the shared DEPs/DETs and we included a supplementary **Figure** **S7** and **Table** **S2.**
c) In the results section, we included a new paragraph '**Validation of the plasma inflammatory proteome’**
d) In the discussion we updated our sentence *“Our study and validation analysis revealed a large number of associations between the glycocalyx disruption marker SDC1 with inflammatory and endothelial proteins.”* **(Line 352-354)**, “*In addition, the cytosolic ubiquitin ligase and antibody receptor TRIM21 was among the top upregulated DEPs and validated proteins.*” **(Line 363-364)**.

**S7 Fig. Shared differentially expressed proteins (DEPs) and previously identified transcripts (DETs) from dengue patients.** DEPs identified in this study were intersected with DETs identified in previous studies. **(A)** Venn chart for the number of shared DEPs/DETs in acute versus convalescent phase or control samples, as well as shared DEPs/DETs per cohort. **(B)** Venn chart for the number of shared DEPs/DETs in severe versus non-severe dengue, as well as shared DEPs/DETs per cohort. Ref: [14-16] as well as Ref: [13,15,23] were combined due to each total genes < 120 genes.

**S2 Table.** Shared differentially expressed proteins (DEPs) and previously identified transcripts (DETs)

| **No** | **Protein** | **Associated Parameters** | **No** | **Protein** | **Associated Parameters** |
| --- | --- | --- | --- | --- | --- |
| *Shared DEPs/DETs in acute versus convalescent phase/controls* | | | | | |
| 1. | ADA | *WBC, SDC1* | 28. | IL32 | *-* |
| 2. | ADGRE2 | *SDC1* | 29. | IL4R | *-* |
| 3. | BCR | *-* | 30. | KYNU | *SDC1* |
| 4. | C1QA | *-* | 31. | LAMP3 | *WBC, SDC1* |
| 5. | CCL20 | *SDC1* | 32. | LAP3 | *SDC1* |
| 6. | CCL3 | *HCT* | 33. | LGALS9 | *-* |
| 7. | CCL4 | *Age, WBC* | 34. | LILRB4 | *WBC* |
| 8. | CD276 | *-* | 35. | LRRN1 | *-* |
| 9. | CD79B | *-* | 36. | LY9 | *HCT* |
| 10. | CLEC4A | *HCT* | 37. | MZB1 | *Sec. Infection* |
| 11. | CRELD2 | *SDC1* | 38. | NBN | *-* |
| 12. | CXCL10 | *-* | 39. | NELL2 | *SDC1* |
| 13. | CXCL8 | *WBC, SDC1* | 40. | PIK3AP1 | *SDC1* |
| 14. | EPHA1 | *-* | 41. | PLAUR | *Age* |
| 15. | FCRL2 | *-* | 42. | PNPT1 | *WBC, SDC1* |
| 16. | FOXO1 | *Sec. infection, WBC, SDC1* | 43. | SAMD9L | *-* |
| 17. | GALNT3 | *WBC* | 44. | SH2D1A | *Sec. Infection, WBC, SDC1* |
| 18. | GZMA | *GBWT* | 45. | SIGLEC1 | *WBC, SDC1* |
| 19. | GZMB | *GBWT* | 46. | SIGLEC10 | *Albumin* |
| 20. | HSPA1A | *ALT, SDC1* | 47. | SLAMF7 | *-* |
| 21. | IFNG | *WBC, SDC1* | 48. | SMPDL3A | *-* |
| 22. | IKBKG | *-* | 49 | TNF | *Age, GBWT* |
| 23. | IL10RA | *-* | 50. | TNFSF10 | *WBC* |
| 24. | IL15 | *WBC, SDC1* | 51. | TRIM21 | *WBC, SDC1* |
| 25. | IL15RA | *-* | 52. | TRIM5 | *-* |
| 26. | IL1R2 | *-* | 53 | VEGFA | *-* |
| 27. | IL1RN | *Age* |  |  |  |
| *Shared DEPs/DETs in severe versus non-severe illness* | | | | | |
| 1. | BACH1 | *SDC1* |  |  |  |
| 2. | CD244 | *PLT* |  |  |  |
| 3. | CXCL10 | *-* |  |  |  |
| 4. | CXCL8 | *WBC, SDC1* |  |  |  |
| 5. | IL10 | *WBC, SDC1* |  |  |  |
| 6. | IL15 | *WBC, SDC1* |  |  |  |
| 7. | IL17C | *ALT* |  |  |  |
| 8. | IL18 | *-* |  |  |  |
| 9. | IL1R2 | *-* |  |  |  |
| 10. | IL1RN | *Age* |  |  |  |
| 11. | KLRD1 | *ALT, SDC1* |  |  |  |
| 12. | LAMP3 | *WBC, SDC1* |  |  |  |
| 13. | NCR1 | *-* |  |  |  |
| 14. | SERPINB8 | *-* |  |  |  |
| 15. | TRAF2 | *-* |  |  |  |

HCT, hematocrit; WBC, white blood cell number; PLT, platelet number; ALT, alanine transferase; SDC1, plasma syndecan-1; Secondary dengue; classified as having a secondary DENV infection, GBWT, gall-bladder wall thickness.

--------------------

**Results**

-Does the analysis presented match the analysis plan?

-Are the results clearly and completely presented?

-Are the figures (Tables, Images) of sufficient quality for clarity?

Reviewer #1: Results are clearly presented and discussed

Response: N/A

Reviewer #2: (No Response)

Response: N/A

--------------------

**Conclusions**

-Are the conclusions supported by the data presented?

-Are the limitations of analysis clearly described?

-Do the authors discuss how these data can be helpful to advance our understanding of the topic under study?

-Is public health relevance addressed?

Reviewer #1: Conclusions are supported by the data. A few minor limitations were not discussed (please see comments below).

Response: N/A

Reviewer #2: (No Response)

Response: N/A

--------------------

**Editorial and Data Presentation Modifications?**

Use this section for editorial suggestions as well as relatively minor modifications of existing data that would enhance clarity. If the only modifications needed are minor and/or editorial, you may wish to recommend “Minor Revision” or “Accept”.

Reviewer #1: Minor revision

Reviewer #2: Minor points:

-As it currently stands, it is hard to infer the significance of the findings considering the rather small fold-change of most of the identified proteins. For instance, in Fig.1, FDR<0.05, red labels: most of these proteins are differentially within 2 and 3-fold change to controls, yet highlighted as up-regulated. Which cut-off criteria was applied to fold-change for hit-calling?

**Response:**

The X-axis of our **Figure 1** represents Log2 Fold-change (the actual fold change values will be 2 exponent the reported values in the X-axis) and the significance cut off was defined using FDR value <0.05. In the case of the protein-protein interaction network, we limited the analysis to the top 50 DEPs with Log2FC > 1.5 (> 3x fold-change) for upregulated proteins (n=42) and Log2FC >1 (>2 fold-change) for downregulated proteins (n=8) **(Line 285-287).**

-lines 365-367: “it inevitable lacks” please correct

**Response:**

We have revised this statement to “*Finally, despite the large number of target proteins in the panel, it inevitably lacks proteins that may be relevant in DENV infection, including type I interferons.*” **(Line 423-425).**

-Mat. And methods, lines 119-134: Olink data processing and analysis: please provide more details. As the assay is not a commonly-used one, the concept behind the DAN barcoding and the data normalization and quantification should be provided.

**Response:**

We thank the reviewer for his suggestions and have included additional sentences within the Olink data processing and analysis paragraph. “*To enhance protein detectability and specificity, this assay utilizes the binding of target proteins by paired oligonucleotide antibody probes, followed by hybridization and amplification using a standard real-time PCR. Protein concentration are reported as normalized protein expression values (NPX), which is an arbitrary unit in a Log2 scale that is calculated from normalized Ct values.*” **(Line 143-147).**

- Figure 1A: How was the statistical significance of the different groups within the PCA calculated? Is this adjusted or unadjusted p-values presented?

**Response:** The statistical significance was obtained from the mean difference between values of PC1 (the axis capturing most variation in the data) from the principal component analysis (PCA) analyzed using Mann-Whitney U test as provided in **Figure 1A** legend (**Line 262-264**).

- Supplementary Figure S4: please rescale the y-axis to display dynamic window of the data more accurately (i.e. p-values compressed towards x-axis).

**Response:** We have adjusted the Y-axis of **S4. Fig** in the new supplementary figures file.

Lastly, we also adjusted several sentences in the discussion “*A number of inflammation-related proteins were associated with the endothelial glycocalyx degradation marker SDC1.*” **(Line 402-403)** and “*Limitations of our study are the fact that the number of participants was limited, and the participants were relatively homogenous in terms of severity of illness.*” (**Line 415-417).**

References

1.	Petrera A, von Toerne C, Behler J, Huth C, Thorand B, Hilgendorff A, et al. Multiplatform Approach for Plasma Proteomics: Complementarity of Olink Proximity Extension Assay Technology to Mass Spectrometry-Based Protein Profiling. J Proteome Res. 2021;20(1):751-62. doi:10.1021/acs.jproteome.0c00641

2.	Han L, Ao X, Lin S, Guan S, Zheng L, Han X, et al. Quantitative Comparative Proteomics Reveal Biomarkers for Dengue Disease Severity. Front Microbiol. 2019;10:2836. doi:10.3389/fmicb.2019.02836

3.	Ray S, Srivastava R, Tripathi K, Vaibhav V, Patankar S, Srivastava S. Serum Proteome Changes in Dengue Virus-Infected Patients from a Dengue-Endemic Area of India: Towards New Molecular Targets? Omics. 2012;16(10):527-36. doi:10.1089/omi.2012.0037

4.	Fragnoud R, Flamand M, Reynier F, Buchy P, Duong V, Pachot A, et al. Differential Proteomic Analysis of Virus-Enriched Fractions Obtained from Plasma Pools of Patients with Dengue Fever or Severe Dengue. BMC Infect Dis. 2015;15:518. doi:10.1186/s12879-015-1271-7

5.	Simmons CP, Popper S, Dolocek C, Chau TN, Griffiths M, Dung NT, et al. Patterns of Host Genome-Wide Gene Transcript Abundance in the Peripheral Blood of Patients with Acute Dengue Hemorrhagic Fever. J Infect Dis. 2007;195(8):1097-107. doi:10.1086/512162

6.	Fink J, Gu F, Ling L, Tolfvenstam T, Olfat F, Chin KC, et al. Host Gene Expression Profiling of Dengue Virus Infection in Cell Lines and Patients. PLoS Negl Trop Dis. 2007;1(2):e86. doi:10.1371/journal.pntd.0000086

7.	Ubol S, Masrinoul P, Chaijaruwanich J, Kalayanarooj S, Charoensirisuthikul T, Kasisith J. Differences in Global Gene Expression in Peripheral Blood Mononuclear Cells Indicate a Significant Role of the Innate Responses in Progression of Dengue Fever but Not Dengue Hemorrhagic Fever. The Journal of Infectious Diseases. 2008;197(10):1459-67. doi:10.1086/587699

8.	de Kruif MD, Setiati TE, Mairuhu AT, Koraka P, Aberson HA, Spek CA, et al. Differential Gene Expression Changes in Children with Severe Dengue Virus Infections. PLoS Negl Trop Dis. 2008;2(4):e215. doi:10.1371/journal.pntd.0000215

9.	Long HT, Hibberd ML, Hien TT, Dung NM, Van Ngoc T, Farrar J, et al. Patterns of Gene Transcript Abundance in the Blood of Children with Severe or Uncomplicated Dengue Highlight Differences in Disease Evolution and Host Response to Dengue Virus Infection. J Infect Dis. 2009;199(4):537-46. doi:10.1086/596507

10.	Nascimento EJ, Braga-Neto U, Calzavara-Silva CE, Gomes AL, Abath FG, Brito CA, et al. Gene Expression Profiling During Early Acute Febrile Stage of Dengue Infection Can Predict the Disease Outcome. PLoS One. 2009;4(11):e7892. doi:10.1371/journal.pone.0007892

11.	Hoang LT, Lynn DJ, Henn M, Birren BW, Lennon NJ, Le PT, et al. The Early Whole-Blood Transcriptional Signature of Dengue Virus and Features Associated with Progression to Dengue Shock Syndrome in Vietnamese Children and Young Adults. J Virol. 2010;84(24):12982-94. doi:10.1128/jvi.01224-10

12.	Devignot S, Sapet C, Duong V, Bergon A, Rihet P, Ong S, et al. Genome-Wide Expression Profiling Deciphers Host Responses Altered During Dengue Shock Syndrome and Reveals the Role of Innate Immunity in Severe Dengue. PLoS One. 2010;5(7):e11671. doi:10.1371/journal.pone.0011671

13.	Sun P, García J, Comach G, Vahey MT, Wang Z, Forshey BM, et al. Sequential Waves of Gene Expression in Patients with Clinically Defined Dengue Illnesses Reveal Subtle Disease Phases and Predict Disease Severity. PLoS Negl Trop Dis. 2013;7(7):e2298. doi:10.1371/journal.pntd.0002298

14.	van de Weg CA, van den Ham HJ, Bijl MA, Anfasa F, Zaaraoui-Boutahar F, Dewi BE, et al. Time since Onset of Disease and Individual Clinical Markers Associate with Transcriptional Changes in Uncomplicated Dengue. PLoS Negl Trop Dis. 2015;9(3):e0003522. doi:10.1371/journal.pntd.0003522

15.	Nikolayeva I, Bost P, Casademont I, Duong V, Koeth F, Prot M, et al. A Blood Rna Signature Detecting Severe Disease in Young Dengue Patients at Hospital Arrival. J Infect Dis. 2018;217(11):1690-8. doi:10.1093/infdis/jiy086

16.	Tolfvenstam T, Lindblom A, Schreiber MJ, Ling L, Chow A, Ooi EE, et al. Characterization of Early Host Responses in Adults with Dengue Disease. BMC Infect Dis. 2011;11:209. doi:10.1186/1471-2334-11-209
